# Supplementary material for: Prediction of HIV status based on socio-behavioural characteristics in East and Southern Africa
Source: PLoS One. 2022 Mar 3;17(3):e0264429. doi: 10.1371/journal.pone.0264429 (PMC8893684; doi:10.1371/journal.pone.0264429)
Supplement: S1 File — (DOCX) [file pone.0264429.s001.docx]

**Supplementary material**

**Individuals**

Data were resampled per country using sample weights from the HIV test results dataset. We excluded individuals whose HIV status was “indeterminate” or “inconclusive” and individuals who reported that they never had sexual intercourse (Table A2).

**Variables**

We removed variables containing more than 30% missing values, variables with no variance and duplicates. We also aggregated some variables together to create new ones and one-hot encoded nominal variables. We finally manually removed an additional 77 non-informative variables for males and 122 for females (e.g. related to metadata or information on how the survey was conducted), resulting in a final dataset of 55,151 males and 69,626 females with 84 and 122 variables respectively (Table A2). Overall, 73 variables were common for both sexes (Table A3).

**Stratification**

The stratification was done based on each sample HIV prevalence to ensure that the percentage of HIV positive individuals in the training, validation and test samples remained similar to the originals.

**MICE imputation**

We imputed missing values on each of the 80% training samples by chained equations (MICE), and applied the same imputation model to the corresponding test and left-out country samples. The regressions on the chained equations have been iterated ten times using the entire set of variables. The imputation has been performed five times and then averaged. For a complete overview, the python script can be found in the project repository at “scripts/data_processing_engineering/imputation.py ”.

**Standardization**

The variables were standardized to a variance of one, ensuring that the penalization scheme was fair to all regressors.

**Models**

Each algorithm was assessed using a stratified 5-fold cross-validation on 50 sets of hyperparameters values, same metrics measured (namely F1, sensitivity, positive predictive value (PPV) and Brier score), same threshold used (0.5), and best model selection based on highest F1 on the test dataset. What differs is the algorithms and their respective objective functions (training loss + regularization) together with the number and type of the hyperparameters.

**Generalized additive models (Logistic GAM)**


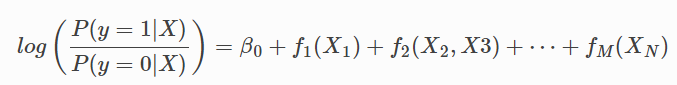


GAM takes the functional form above with in our case the intercept set to zero. Since the scale of the Binomial distribution is known, our gridsearch minimizes an Un-Biased Risk Estimator (UBRE) objective:
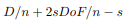
where D is the deviance, n the number of data, s the scale parameter (equal to 1 in our case) and DoF the effective degrees of freedom of the model. The feature functions are penalized B splines (P spline) with 20 basis functions for each by default. The smoothing hyperparameter lambda is drawn from a continuous uniform distribution between exp(-3) and exp(3) for each of the spline functions.

**Penalized Logistic Regression (Elastic Net)**

**
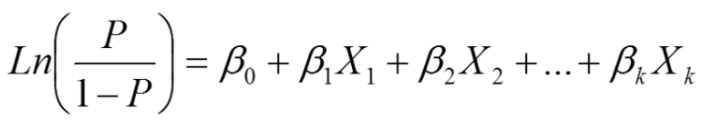
**

Logistic regression takes the above functional form with in our case the Intercept set to zero. The two hyperparameters of the loss function below were evenly distributed on a logarithmic space (C: logspace(-9, 9), l1ratio: logspace(-9, 0)):


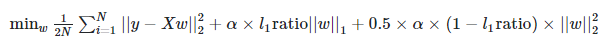


The parameter C is the inverse of the regularization strength alpha and l1ratio is the Elastic-Net mixing regularization hyperparameter where l1ratio=0 is equivalent to using a l2 penalty and l1ratio=1 equivalent to using a l1 penalty.

**Support Vector Classifier (SVC)**

The radial basis function kernel has been used for this learning algorithm. The parameter C (evenly distributed on a logarithmic space (logspace(-9, 9)) is the regularization hyperparameter, similar to the one of Elastic Net (i.e. the strength of the regularization is inversely proportional to C) of the below loss function. The penalty is a squared l2 penalty:


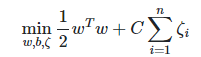


**Extreme Gradient Boosting (XGBoost)**

Nine hyperparameters were included into the random grid search. The details of each parameter space are:


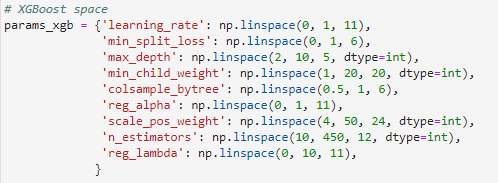


The objective function is:
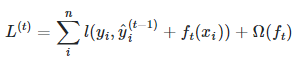


where in our case, l is the log likelihood of the Bernoulli distribution (i.e. log loss). For a more detailed overview of the regularization term and all the hyperparameters you can refer to the following article by Chen & Guestrin: <https://arxiv.org/pdf/1603.02754.pdf>.
