# Supplementary material for: Prediction of HIV status based on socio-behavioural characteristics in East and Southern Africa
Source: PLoS One. 2022 Mar 3;17(3):e0264429. doi: 10.1371/journal.pone.0264429 (PMC8893684; doi:10.1371/journal.pone.0264429)

**Fig S1: Values of the F1 score and the Brier score for each of the 50 sets of parameters per algorithm (female ex-Zambia dataset)**


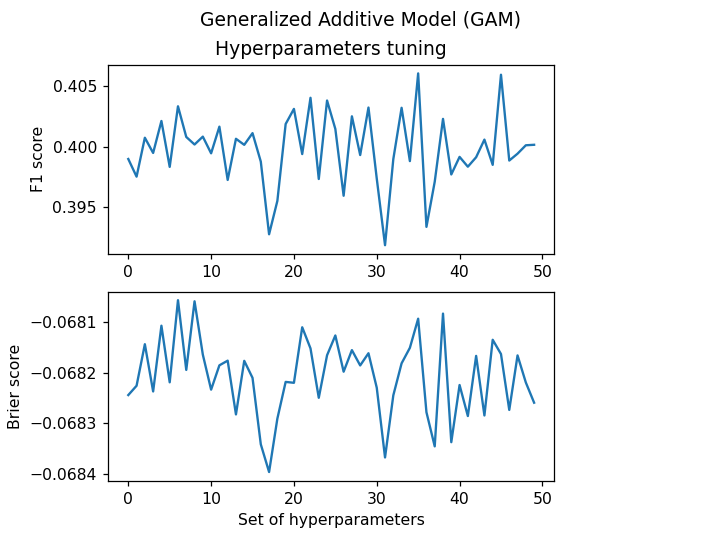

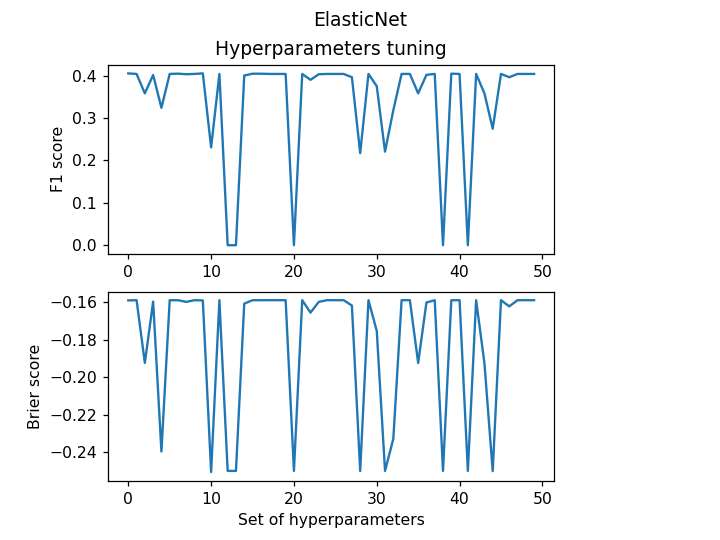

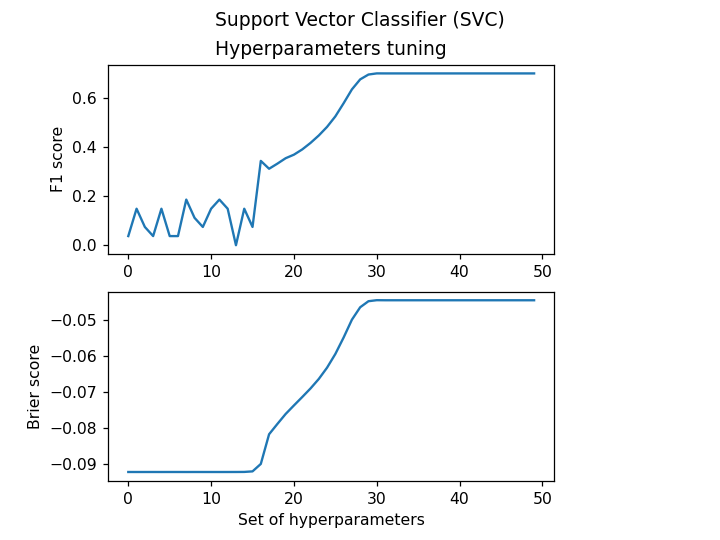

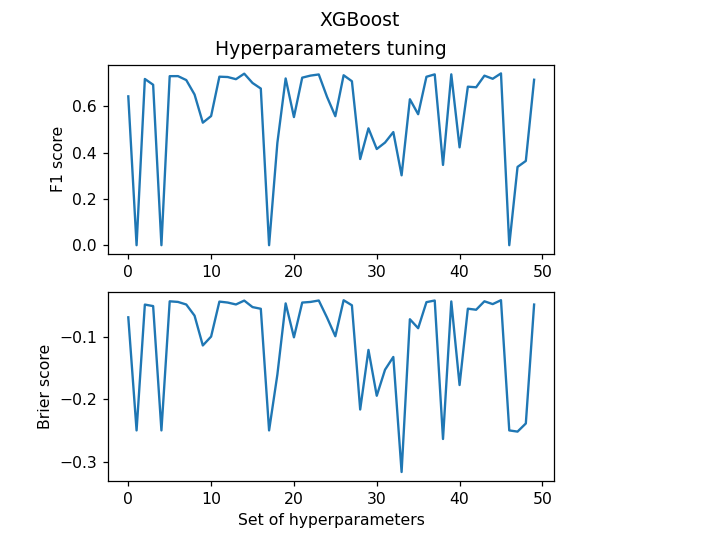

Supplement: S1 Fig — (DOCX) [file pone.0264429.s002.docx]
