# Supplementary material for: Prediction of HIV status based on socio-behavioural characteristics in East and Southern Africa
Source: PLoS One. 2022 Mar 3;17(3):e0264429. doi: 10.1371/journal.pone.0264429 (PMC8893684; doi:10.1371/journal.pone.0264429)
Supplement: S1 Table — (DOCX) [file pone.0264429.s003.docx]

**Table S1: List of the Demographic and Health Surveys (DHS) Survey Year**

| **Survey** | **Year** | **Country** | **Male** | | **Female** | |
| --- | --- | --- | --- | --- | --- | --- |
|  |  |  | Individuals | Variables | Individuals | Variables |
| Standard DHS VII | 2015-2016 | Angola | 5,684 | 592 | 14,379 | 5,330 |
| Standard DHS VII | 2016-2017 | Burundi | 7,552 | 763 | 17,269 | 5,021 |
| Standard DHS VII | 2016 | Ethiopia | 12,688 | 590 | 15,683 | 5,695 |
| Standard DHS VII | 2014 | Lesotho | 2,931 | 627 | 6,621 | 3,748 |
| Standard DHS VII | 2015-2016 | Malawi | 7,478 | 571 | 24,562 | 4,934 |
| Standard AIS DHS VII | 2015 | Mozambique | 5,283 | 796 | 7,749 | 4,861 |
| Standard DHS VI | 2013 | Namibia | 4,481 | 641 | 10,018 | 4,180 |
| Standard DHS VII | 2014-2015 | Rwanda | 6,217 | 702 | 13,497 | 4,572 |
| Standard DHS VI | 2013-2014 | Zambia | 14,773 | 864 | 16,411 | 4,266 |
| Standard DHS VII | 2015 | Zimbabwe | 8,396 | 605 | 9,955 | 4,940 |
|  |  | **Total** | 75,483 |  | 136,144 |  |
