# Supplementary material for: Prediction of HIV status based on socio-behavioural characteristics in East and Southern Africa
Source: PLoS One. 2022 Mar 3;17(3):e0264429. doi: 10.1371/journal.pone.0264429 (PMC8893684; doi:10.1371/journal.pone.0264429)
Supplement: S2 Table — (DOCX) [file pone.0264429.s004.docx]

Table S2: Data Processing

| **Processing** | **Men** | | **Women** | |
| --- | --- | --- | --- | --- |
|  | # of individuals | # of variables | # of individuals | # of variables |
| **Reduction of variables** |  | 527 |  | 3,213 |
| 30% of missing values |  | 203 |  | 310 |
| no variance |  | 178 |  | 270 |
| duplicate variables |  | 173 |  | 261 |
| aggregation and removal |  | 84 |  | 122 |
| **Reduction of individuals** | 68,979 |  | 83,910 |  |
| inconclusive HIV testing | 68,669 |  | 83,678 |  |
| no sexual intercourse | 55,151 |  | 69,626 |  |
| **Final datasets** | **55,151** | **84** | **69,626** | **122** |
|  |  |  |  |  |
| 30% of missing values: removing variables that have more than 30% of missing values | | | |  |
| no variance: removing constant variables | |  |  |  |
| duplicate variables: removing duplicated variables | |  |  |  |
| aggregation and removal: removing uninformative variables, aggregating others and creating dummy ones | | | | |
| inconclusive HIV testing: removing individuals with inconclusive HIV test | | |  |  |
| no sexual intercourse: removing individuals with no sexual intercourse | | |  |  |
