# Supplementary material for: Prediction of HIV status based on socio-behavioural characteristics in East and Southern Africa
Source: PLoS One. 2022 Mar 3;17(3):e0264429. doi: 10.1371/journal.pone.0264429 (PMC8893684; doi:10.1371/journal.pone.0264429)
Supplement: S3 Table — (DOCX) [file pone.0264429.s005.docx]

| **Table S3i: Summary statistics of the observed and imputed data for the incomplete variables in the male test dataset** | | | | | | | | | | | |
| --- | --- | --- | --- | --- | --- | --- | --- | --- | --- | --- | --- |
|  | **Observed** | | | | |  | **Imputed** | | | | |
|  | **N** | **Mean** | **Std** | **Min** | **Max** |  | **N** | **Mean** | **Std** | **Min** | **Max** |
| Years lived in place of residence | 8,143 | 21.7 | 15.4 | 0 | 59 |  | 2,888 | 22.6 | 10.5 | 0 | 58 |
| Age of most recent partner | 9,576 | 28.9 | 10.0 | 10 | 100 |  | 1,455 | 22.3 | 8.7 | 10 | 54 |
| Type of earnings from respondent's work | 9,640 | 2.1 | 1.2 | 0 | 3 |  | 1,391 | 2.3 | 0.6 | 1 | 3 |
| Employment all year/seasonal | 9,645 | 2.4 | 0.8 | 0 | 3 |  | 1,386 | 1.6 | 0.5 | 0 | 3 |
| Women who use contraception become promiscuous | 9,687 | 0.3 | 0.5 | 0 | 1 |  | 1,344 | 0.3 | 0.5 | 0 | 1 |
| Times in last 12 months had sex with most recent partner | 9,726 | 89.8 | 63.5 | 1 | 146 |  | 1,305 | 14.6 | 30.5 | 1 | 146 |
| Condom used during last sex with most recent partner | 9,821 | 0.2 | 0.4 | 0 | 1 |  | 1,210 | 0.2 | 0.4 | 0 | 1 |
| Relationship with most recent sex partner | 9,821 | 0.7 | 1.0 | 0 | 5 |  | 1,210 | 1.8 | 0.7 | 0 | 3 |
| Contraception is woman's business, man should not worry | 9,835 | 0.2 | 0.4 | 0 | 1 |  | 1,196 | 0.3 | 0.4 | 0 | 1 |
| Ideal number of boys | 9,893 | 2.3 | 1.7 | 0 | 25 |  | 1,138 | 2.6 | 0.5 | 2 | 4 |
| Ideal number of girls | 9,893 | 1.9 | 1.5 | 0 | 25 |  | 1,138 | 1.9 | 0.3 | 1 | 3 |
| Ideal number of either sex | 9,893 | 0.4 | 1.4 | 0 | 20 |  | 1,138 | 0.3 | 0.5 | 0 | 6 |
| Ideal number of children | 9,932 | 4.6 | 2.8 | 0 | 50 |  | 1,099 | 4.9 | 0.4 | 4 | 6 |
| Drugs to avoid HIV transmission to baby during pregnancy | 10,060 | 0.8 | 0.4 | 0 | 1 |  | 971 | 0.7 | 0.4 | 0 | 1 |
| Covered by health insurance | 10,114 | 0.1 | 0.3 | 0 | 1 |  | 917 | 0.0 | 0.1 | 0 | 1 |
| Discussed Family Planning with health worker in last few months | 10,119 | 0.2 | 0.4 | 0 | 1 |  | 912 | 0.0 | 0.2 | 0 | 1 |
| Fertility preference | 10,127 | 2.1 | 0.8 | 0 | 3 |  | 904 | 2.0 | 0.7 | 0 | 3 |
| Heard family planning in newspaper/magazine last few months | 10,131 | 0.2 | 0.4 | 0 | 1 |  | 900 | 0.1 | 0.2 | 0 | 1 |
| Knowledge of ovulatory cycle_after period ended | 10,131 | 0.3 | 0.5 | 0 | 1 |  | 900 | 0.0 | 0.0 | 0 | 0 |
| Knowledge of ovulatory cycle_at any time | 10,131 | 0.1 | 0.3 | 0 | 1 |  | 900 | 0.0 | 0.0 | 0 | 0 |
| Knowledge of ovulatory cycle_before period begins | 10,131 | 0.2 | 0.4 | 0 | 1 |  | 900 | 0.0 | 0.0 | 0 | 0 |
| Knowledge of ovulatory cycle_don't know | 10,131 | 0.1 | 0.3 | 0 | 1 |  | 900 | 0.0 | 0.0 | 0 | 0 |
| Knowledge of ovulatory cycle_during her period | 10,131 | 0.0 | 0.2 | 0 | 1 |  | 900 | 0.0 | 0.0 | 0 | 0 |
| Knowledge of ovulatory cycle_middle of the cycle | 10,131 | 0.1 | 0.3 | 0 | 1 |  | 900 | 0.6 | 0.5 | 0 | 1 |
| Knowledge of ovulatory cycle_other | 10,131 | 0.0 | 0.0 | 0 | 1 |  | 900 | 0.4 | 0.5 | 0 | 1 |
| Heard family planning on TV last few months | 10,133 | 0.2 | 0.4 | 0 | 1 |  | 898 | 0.1 | 0.3 | 0 | 1 |
| Knowledge of any contraceptive method | 10,135 | 3.0 | 0.2 | 0 | 3 |  | 896 | 3.0 | 0.2 | 2 | 3 |
| Heard family planning on radio last few months | 10,135 | 0.5 | 0.5 | 0 | 1 |  | 896 | 0.3 | 0.5 | 0 | 1 |
| Literacy | 10,172 | 1.5 | 0.8 | 0 | 2 |  | 859 | 1.3 | 0.6 | 0 | 2 |
| Know a place to get HIV test | 10,319 | 0.9 | 0.2 | 0 | 1 |  | 712 | 1.0 | 0.2 | 0 | 1 |
| Total lifetime number of sex partners | 10,673 | 6.4 | 16.5 | 1 | 146 |  | 358 | 12.5 | 10.4 | 1 | 64 |
| Occupation_agricultural | 10,826 | 0.4 | 0.5 | 0 | 1 |  | 205 | 0.1 | 0.3 | 0 | 1 |
| Occupation_clerical | 10,826 | 0.0 | 0.1 | 0 | 1 |  | 205 | 0.0 | 0.1 | 0 | 1 |
| Occupation_household and domestic | 10,826 | 0.0 | 0.1 | 0 | 1 |  | 205 | 0.0 | 0.0 | 0 | 0 |
| Occupation_manual | 10,826 | 0.2 | 0.4 | 0 | 1 |  | 205 | 0.0 | 0.1 | 0 | 1 |
| Occupation_not working | 10,826 | 0.1 | 0.3 | 0 | 1 |  | 205 | 0.0 | 0.0 | 0 | 0 |
| Occupation_other | 10,826 | 0.0 | 0.1 | 0 | 1 |  | 205 | 0.0 | 0.0 | 0 | 0 |
| Occupation_professional/technical/managerial | 10,826 | 0.1 | 0.3 | 0 | 1 |  | 205 | 0.5 | 0.5 | 0 | 1 |
| Occupation_sales | 10,826 | 0.1 | 0.3 | 0 | 1 |  | 205 | 0.4 | 0.5 | 0 | 1 |
| Occupation_services | 10,826 | 0.1 | 0.2 | 0 | 1 |  | 205 | 0.0 | 0.0 | 0 | 0 |
| Age at first sex (imputed) | 10,833 | 18.6 | 4.3 | 6 | 47 |  | 198 | 20.1 | 2.7 | 13 | 28 |
| Have ever paid anyone in exchange for sex | 10,841 | 0.1 | 0.3 | 0 | 1 |  | 190 | 0.0 | 0.1 | 0 | 1 |
| Paid for sex in last 12 months | 10,842 | 0.0 | 0.2 | 0 | 1 |  | 189 | 0.0 | 0.0 | 0 | 0 |
| Wife justified refusing sex: husband has other women | 10,872 | 0.8 | 0.4 | 0 | 1 |  | 159 | 0.5 | 0.5 | 0 | 1 |
| Ways of transmission from mother to child | 10,909 | 0.5 | 0.5 | 0 | 1 |  | 122 | 0.6 | 0.5 | 0 | 1 |
| Would buy vegetables from vendor with HIV | 10,910 | 0.8 | 0.4 | 0 | 1 |  | 121 | 0.5 | 0.5 | 0 | 1 |
| Reduce risk of getting HIV | 10,911 | 1.0 | 0.1 | 0 | 1 |  | 120 | 1.0 | 0.0 | 1 | 1 |
| Number of women fathered children with | 10,953 | 0.9 | 0.8 | 0 | 15 |  | 78 | 1.8 | 0.5 | 1 | 3 |
| Sons at home | 10,958 | 1.0 | 1.3 | 0 | 9 |  | 73 | 1.5 | 0.9 | 0 | 4 |
| Daughters at home | 10,958 | 0.9 | 1.2 | 0 | 12 |  | 73 | 1.4 | 0.7 | 0 | 3 |
| Sons elsewhere | 10,958 | 0.4 | 0.9 | 0 | 23 |  | 73 | 1.4 | 0.6 | 0 | 3 |
| Daughters elsewhere | 10,958 | 0.4 | 0.9 | 0 | 13 |  | 73 | 1.5 | 0.6 | 0 | 3 |
| Sons who have died | 10,958 | 0.2 | 0.6 | 0 | 12 |  | 73 | 0.5 | 0.5 | 0 | 1 |
| Daughters who have died | 10,958 | 0.2 | 0.5 | 0 | 9 |  | 73 | 0.4 | 0.5 | 0 | 1 |
| Had any STI in last 12 months | 10,988 | 0.0 | 0.2 | 0 | 1 |  | 43 | 0.0 | 0.2 | 0 | 1 |
| Current contraceptive method | 10,990 | 0.2 | 0.4 | 0 | 1 |  | 41 | 0.0 | 0.2 | 0 | 1 |
| Current contraceptive by method type | 10,990 | 1.4 | 1.5 | 0 | 3 |  | 41 | 0.5 | 0.6 | 0 | 2 |
| Cluster altitude in meters | 10,996 | 1,243.9 | 609.1 | 2 | 3,455 |  | 35 | 798.2 | 313.6 | 80 | 1,303 |
| Had genital sore/ulcer in last 12 months | 10,998 | 0.0 | 0.2 | 0 | 1 |  | 33 | 0.0 | 0.0 | 0 | 0 |
| Had genital discharge in last 12 months | 11,001 | 0.0 | 0.2 | 0 | 1 |  | 30 | 0.0 | 0.2 | 0 | 1 |
| Times away from home in last 12 months | 11,010 | 4.7 | 17.1 | 0 | 146 |  | 21 | 5.4 | 6.0 | 0 | 20 |
| Number of sex partners, including spouse, in last 12 months | 11,012 | 1.1 | 1.2 | 0 | 61 |  | 19 | 2.0 | 1.3 | 0 | 5 |
| Respondent circumcised | 11,015 | 0.5 | 0.5 | 0 | 1 |  | 16 | 0.3 | 0.5 | 0 | 1 |
| Heard about other STIs | 11,015 | 0.8 | 0.4 | 0 | 1 |  | 16 | 0.9 | 0.3 | 0 | 1 |
| Religion_catholicism | 11,016 | 0.3 | 0.4 | 0 | 1 |  | 15 | 0.7 | 0.5 | 0 | 1 |
| Religion_islam | 11,016 | 0.1 | 0.3 | 0 | 1 |  | 15 | 0.0 | 0.0 | 0 | 0 |
| Religion_no religion | 11,016 | 0.1 | 0.2 | 0 | 1 |  | 15 | 0.0 | 0.0 | 0 | 0 |
| Religion_other | 11,016 | 0.1 | 0.2 | 0 | 1 |  | 15 | 0.0 | 0.0 | 0 | 0 |
| Religion_other christian | 11,016 | 0.1 | 0.3 | 0 | 1 |  | 15 | 0.0 | 0.0 | 0 | 0 |
| Religion_protestantism | 11,016 | 0.4 | 0.5 | 0 | 1 |  | 15 | 0.3 | 0.5 | 0 | 1 |
| Recent sexual activity | 11,017 | 0.7 | 0.5 | 0 | 1 |  | 14 | 0.4 | 0.5 | 0 | 1 |
| Time since last sex (in days) | 11,019 | 153.5 | 581.4 | 0 | 10,585 |  | 12 | 363.2 | 322.2 | 0 | 846 |
| Age of household head | 11,021 | 41.1 | 13.1 | 16 | 100 |  | 10 | 50.0 | 6.1 | 43 | 61 |
| Owns land alone or jointly | 11,021 | 0.9 | 1.0 | 0 | 3 |  | 10 | 0.9 | 0.7 | 0 | 2 |
| Owns a house alone or jointly | 11,026 | 1.0 | 1.0 | 0 | 3 |  | 5 | 1.8 | 1.1 | 0 | 3 |
| Frequency of listening to radio | 11,027 | 1.4 | 1.0 | 0 | 3 |  | 4 | 2.0 | 0.0 | 2 | 2 |
| Number of injections in last 12 months | 11,027 | 0.8 | 4.9 | 0 | 146 |  | 4 | 1.5 | 1.9 | 0 | 4 |
| Frequency of watching television | 11,028 | 0.9 | 1.1 | 0 | 3 |  | 3 | 1.0 | 1.0 | 0 | 2 |
| Currently working | 11,028 | 0.8 | 0.4 | 0 | 1 |  | 3 | 0.0 | 0.0 | 0 | 0 |
| Total number of years of education | 11,029 | 6.8 | 4.4 | 0 | 24 |  | 2 | 9.0 | 0.0 | 9 | 9 |
| Usual resident or visitor | 11,029 | 1.0 | 0.1 | 0 | 1 |  | 2 | 1.0 | 0.0 | 1 | 1 |
| Wife justified asking husband to use condom if he has STI | 11,029 | 0.9 | 0.3 | 0 | 1 |  | 2 | 1.0 | 0.0 | 1 | 1 |
| Highest educational level | 11,030 | 1.4 | 0.8 | 0 | 3 |  | 1 | 2.0 |  | 2 | 2 |
| Frequency of reading newspaper or magazine | 11,030 | 0.6 | 0.9 | 0 | 3 |  | 1 | 2.0 |  | 2 | 2 |
| Respondent worked in last 7 days | 11,030 | 1.7 | 0.7 | 0 | 2 |  | 1 | 0.0 |  | 0 | 0 |
| Ever been tested for HIV | 11,030 | 0.7 | 0.5 | 0 | 1 |  | 1 | 0.7 |  | 1 | 1 |
| Relationship to household head_brother/sister | 11,030 | 0.0 | 0.2 | 0 | 1 |  | 1 | 0.0 |  | 0 | 0 |
| Relationship to household head_children-in-law | 11,030 | 0.0 | 0.1 | 0 | 1 |  | 1 | 0.0 |  | 0 | 0 |
| Relationship to household head_head | 11,030 | 0.7 | 0.5 | 0 | 1 |  | 1 | 1.0 |  | 1 | 1 |
| Relationship to household head_husband/wife | 11,030 | 0.0 | 0.1 | 0 | 1 |  | 1 | 0.0 |  | 0 | 0 |
| Relationship to household head_not related | 11,030 | 0.0 | 0.1 | 0 | 1 |  | 1 | 0.0 |  | 0 | 0 |
| Relationship to household head_other relative | 11,030 | 0.1 | 0.3 | 0 | 1 |  | 1 | 0.0 |  | 0 | 0 |
| Relationship to household head_parent | 11,030 | 0.0 | 0.0 | 0 | 1 |  | 1 | 0.0 |  | 0 | 0 |
| Relationship to household head_parent-in-law | 11,030 | 0.0 | 0.0 | 0 | 1 |  | 1 | 0.0 |  | 0 | 0 |
| Relationship to household head_son/daughter | 11,030 | 0.2 | 0.4 | 0 | 1 |  | 1 | 0.0 |  | 0 | 0 |
| Beating justified | 11,030 | 0.3 | 0.4 | 0 | 1 |  | 1 | 0.3 |  | 0 | 0 |
