# Supplementary material for: Prediction of HIV status based on socio-behavioural characteristics in East and Southern Africa
Source: PLoS One. 2022 Mar 3;17(3):e0264429. doi: 10.1371/journal.pone.0264429 (PMC8893684; doi:10.1371/journal.pone.0264429)
Supplement: S4 Table — (DOCX) [file pone.0264429.s006.docx]

| **Table S3ii: Summary statistics of the observed and imputed data for the incomplete variables in the female test dataset** | | | | | | | | | | | |
| --- | --- | --- | --- | --- | --- | --- | --- | --- | --- | --- | --- |
|  | **Observed** | | | | |  | **Imputed** | | | | |
|  | **N** | **Mean** | **Std** | **Min** | **Max** |  | **N** | **Mean** | **Std** | **Min** | **Max** |
| Years lived in place of residence | 10,157 | 18.3 | 13.6 | 0 | 49 |  | 3,769 | 20.8 | 9.9 | 0 | 55 |
| Rohrer's index | 10,432 | 1,459.7 | 291.0 | 801 | 5,773 |  | 3,494 | 1,474.2 | 189.8 | 800 | 2,247 |
| Toilet facilities shared with other households | 10,933 | 0.3 | 0.5 | 0 | 1 |  | 2,993 | 0.2 | 0.4 | 0 | 1 |
| Type of mosquito bed net(s) slept under last night | 11,116 | 1.1 | 1.4 | 0 | 3 |  | 2,810 | 1.1 | 0.7 | 0 | 3 |
| Have mosquito bed net for sleeping | 11,116 | 0.6 | 0.5 | 0 | 1 |  | 2,810 | 0.7 | 0.5 | 0 | 1 |
| Respondent slept under mosquito bed net | 11,116 | 0.4 | 0.5 | 0 | 1 |  | 2,810 | 0.3 | 0.5 | 0 | 1 |
| Age of most recent partner | 11,298 | 36.3 | 11.2 | 10 | 100 |  | 2,628 | 38.4 | 12.4 | 10 | 73 |
| Visited by fieldworker in last 12 months | 11,336 | 0.1 | 0.4 | 0 | 1 |  | 2,590 | 0.0 | 0.1 | 0 | 1 |
| Times in last 12 months had sex with most recent partner | 11,519 | 101.8 | 58.8 | 1 | 146 |  | 2,407 | 18.5 | 31.9 | 1 | 146 |
| Times away from home in last 12 months | 11,709 | 2.0 | 9.0 | 0 | 146 |  | 2,217 | 1.6 | 2.4 | 0 | 15 |
| Relationship with most recent sex partner | 11,742 | 0.5 | 0.8 | 0 | 5 |  | 2,184 | 0.8 | 0.8 | 0 | 3 |
| Condom used during last sex with most recent partner | 11,743 | 0.1 | 0.3 | 0 | 1 |  | 2,183 | 0.1 | 0.2 | 0 | 1 |
| Number of unions | 11,880 | 0.2 | 0.4 | 0 | 1 |  | 2,046 | 0.0 | 0.1 | 0 | 1 |
| Age at first cohabitation | 11,887 | 18.8 | 4.6 | 8 | 63 |  | 2,039 | 22.2 | 5.4 | 15 | 46 |
| Years since first cohabitation | 11,887 | 13.0 | 9.1 | 0 | 46 |  | 2,039 | 1.2 | 2.7 | 0 | 18 |
| Ideal number of boys | 12,032 | 1.9 | 1.3 | 0 | 20 |  | 1,894 | 2.0 | 0.5 | 0 | 5 |
| Ideal number of girls | 12,032 | 1.9 | 1.2 | 0 | 15 |  | 1,894 | 1.9 | 0.5 | 0 | 4 |
| Ideal number of either sex | 12,032 | 0.5 | 1.5 | 0 | 25 |  | 1,894 | 0.5 | 0.6 | 0 | 5 |
| Ideal number of children | 12,047 | 4.2 | 2.1 | 0 | 30 |  | 1,879 | 4.2 | 0.5 | 2 | 7 |
| Owns land alone or jointly | 12,347 | 0.7 | 0.9 | 0 | 3 |  | 1,579 | 0.6 | 0.6 | 0 | 2 |
| Owns a house alone or jointly | 12,360 | 0.8 | 0.9 | 0 | 3 |  | 1,566 | 0.7 | 0.6 | 0 | 2 |
| Drugs to avoid HIV transmission to baby during pregnancy | 12,427 | 0.9 | 0.3 | 0 | 1 |  | 1,499 | 0.6 | 0.5 | 0 | 1 |
| Knowledge of ovulatory cycle_after period ended | 12,491 | 0.4 | 0.5 | 0 | 1 |  | 1,435 | 0.1 | 0.3 | 0 | 1 |
| Knowledge of ovulatory cycle_at any time | 12,491 | 0.1 | 0.3 | 0 | 1 |  | 1,435 | 0.2 | 0.4 | 0 | 1 |
| Knowledge of ovulatory cycle_before period begins | 12,491 | 0.1 | 0.3 | 0 | 1 |  | 1,435 | 0.1 | 0.2 | 0 | 1 |
| Knowledge of ovulatory cycle_don't know | 12,491 | 0.1 | 0.3 | 0 | 1 |  | 1,435 | 0.3 | 0.5 | 0 | 1 |
| Knowledge of ovulatory cycle_during her period | 12,491 | 0.0 | 0.2 | 0 | 1 |  | 1,435 | 0.1 | 0.3 | 0 | 1 |
| Knowledge of ovulatory cycle_middle of the cycle | 12,491 | 0.2 | 0.4 | 0 | 1 |  | 1,435 | 0.1 | 0.3 | 0 | 1 |
| Knowledge of ovulatory cycle_other | 12,491 | 0.0 | 0.0 | 0 | 1 |  | 1,435 | 0.1 | 0.3 | 0 | 1 |
| Heard family planning in newspaper/magazine last few months | 12,492 | 0.1 | 0.3 | 0 | 1 |  | 1,434 | 0.0 | 0.1 | 0 | 1 |
| Visited health facility last 12 months | 12,499 | 0.6 | 0.5 | 0 | 1 |  | 1,427 | 0.3 | 0.5 | 0 | 1 |
| Ever had a terminated pregnancy | 12,502 | 0.1 | 0.3 | 0 | 1 |  | 1,424 | 0.0 | 0.2 | 0 | 1 |
| Index last child prior to maternity-health (calendar) | 12,502 | 1.3 | 1.2 | 0 | 7 |  | 1,424 | 1.1 | 0.9 | 0 | 4 |
| Knowledge of any contraceptive method | 12,502 | 2.9 | 0.5 | 0 | 3 |  | 1,424 | 2.9 | 0.4 | 1 | 3 |
| Pattern of contraceptive use | 12,502 | 1.6 | 1.3 | 0 | 3 |  | 1,424 | 0.6 | 0.7 | 0 | 3 |
| Contraceptive use and intention | 12,502 | 1.5 | 1.2 | 0 | 3 |  | 1,424 | 1.1 | 1.2 | 0 | 3 |
| Heard family planning on radio last few months | 12,502 | 0.3 | 0.5 | 0 | 1 |  | 1,424 | 0.1 | 0.3 | 0 | 1 |
| Heard family planning on TV last few months | 12,502 | 0.2 | 0.4 | 0 | 1 |  | 1,424 | 0.1 | 0.3 | 0 | 1 |
| Literacy | 12,589 | 1.2 | 0.9 | 0 | 2 |  | 1,337 | 1.1 | 0.7 | 0 | 2 |
| Heard of oral rehydration | 12,619 | 0.9 | 0.5 | 0 | 2 |  | 1,307 | 0.9 | 0.3 | 0 | 2 |
| Getting medical help for self: getting money needed for treatment | 12,642 | 0.5 | 0.5 | 0 | 1 |  | 1,284 | 0.6 | 0.5 | 0 | 1 |
| Getting medical help for self: distance to health facility | 12,643 | 0.4 | 0.5 | 0 | 1 |  | 1,283 | 0.5 | 0.5 | 0 | 1 |
| Getting medical help for self: not wanting to go alone | 12,643 | 0.2 | 0.4 | 0 | 1 |  | 1,283 | 0.2 | 0.4 | 0 | 1 |
| Getting medical help for self: getting permission to go | 12,644 | 0.1 | 0.3 | 0 | 1 |  | 1,282 | 0.0 | 0.1 | 0 | 1 |
| Currently amenorrheic | 12,646 | 0.2 | 0.4 | 0 | 1 |  | 1,280 | 0.1 | 0.3 | 0 | 1 |
| Currently abstaining | 12,646 | 0.1 | 0.3 | 0 | 1 |  | 1,280 | 0.0 | 0.2 | 0 | 1 |
| Does not use cigarettes and tobacco | 12,646 | 1.0 | 0.2 | 0 | 1 |  | 1,280 | 1.0 | 0.0 | 0 | 1 |
| Covered by health insurance | 12,905 | 0.1 | 0.3 | 0 | 1 |  | 1,021 | 0.0 | 0.1 | 0 | 1 |
| Know a place to get HIV test | 13,396 | 0.9 | 0.3 | 0 | 1 |  | 530 | 0.7 | 0.5 | 0 | 1 |
| Wife justified refusing sex: husband has other women | 13,403 | 0.7 | 0.5 | 0 | 1 |  | 523 | 0.5 | 0.5 | 0 | 1 |
| Would buy vegetables from vendor with HIV | 13,403 | 0.7 | 0.4 | 0 | 1 |  | 523 | 0.3 | 0.4 | 0 | 1 |
| Reduce risk of getting HIV | 13,406 | 0.6 | 0.5 | 0 | 1 |  | 520 | 0.9 | 0.2 | 0 | 1 |
| Ways of transmission from mother to child | 13,406 | 0.4 | 0.5 | 0 | 1 |  | 520 | 0.5 | 0.5 | 0 | 1 |
| Age at first sex (imputed) | 13,442 | 17.4 | 3.4 | 6 | 63 |  | 484 | 16.4 | 2.5 | 10 | 24 |
| Time to get to water source | 13,443 | 25.5 | 45.5 | 0 | 900 |  | 483 | 25.8 | 20.1 | 0 | 108 |
| Occupation_agricultural | 13,589 | 0.3 | 0.5 | 0 | 1 |  | 337 | 0.5 | 0.5 | 0 | 1 |
| Occupation_clerical | 13,589 | 0.0 | 0.1 | 0 | 1 |  | 337 | 0.0 | 0.0 | 0 | 0 |
| Occupation_household and domestic | 13,589 | 0.0 | 0.1 | 0 | 1 |  | 337 | 0.0 | 0.0 | 0 | 0 |
| Occupation_manual | 13,589 | 0.1 | 0.2 | 0 | 1 |  | 337 | 0.0 | 0.0 | 0 | 0 |
| Occupation_not working | 13,589 | 0.4 | 0.5 | 0 | 1 |  | 337 | 0.0 | 0.0 | 0 | 0 |
| Occupation_other | 13,589 | 0.0 | 0.1 | 0 | 1 |  | 337 | 0.0 | 0.0 | 0 | 0 |
| Occupation_professional/technical/managerial | 13,589 | 0.0 | 0.2 | 0 | 1 |  | 337 | 0.0 | 0.2 | 0 | 1 |
| Occupation_sales | 13,589 | 0.2 | 0.4 | 0 | 1 |  | 337 | 0.0 | 0.2 | 0 | 1 |
| Occupation_services | 13,589 | 0.0 | 0.2 | 0 | 1 |  | 337 | 0.4 | 0.5 | 0 | 1 |
| Fertility preference | 13,593 | 2.1 | 1.0 | 0 | 3 |  | 333 | 1.5 | 1.3 | 0 | 3 |
| Unmet need for contraception_infecund, menopausal | 13,609 | 0.1 | 0.3 | 0 | 1 |  | 317 | 0.2 | 0.4 | 0 | 1 |
| Unmet need for contraception_no unmet need | 13,609 | 0.2 | 0.4 | 0 | 1 |  | 317 | 0.2 | 0.4 | 0 | 1 |
| Unmet need for contraception_not married and no sex in last 30 days | 13,609 | 0.1 | 0.3 | 0 | 1 |  | 317 | 0.0 | 0.0 | 0 | 0 |
| Unmet need for contraception_unmet need for limiting | 13,609 | 0.1 | 0.3 | 0 | 1 |  | 317 | 0.0 | 0.0 | 0 | 0 |
| Unmet need for contraception_unmet need for spacing | 13,609 | 0.1 | 0.3 | 0 | 1 |  | 317 | 0.3 | 0.5 | 0 | 1 |
| Unmet need for contraception_using for limiting | 13,609 | 0.2 | 0.4 | 0 | 1 |  | 317 | 0.3 | 0.5 | 0 | 1 |
| Unmet need for contraception_using for spacing | 13,609 | 0.2 | 0.4 | 0 | 1 |  | 317 | 0.0 | 0.1 | 0 | 1 |
| Sons at home | 13,612 | 1.1 | 1.2 | 0 | 7 |  | 314 | 1.2 | 0.4 | 0 | 2 |
| Daughters at home | 13,612 | 1.1 | 1.2 | 0 | 7 |  | 314 | 1.0 | 0.4 | 0 | 2 |
| Sons elsewhere | 13,612 | 0.3 | 0.6 | 0 | 7 |  | 314 | 0.3 | 0.4 | 0 | 1 |
| Daughters elsewhere | 13,612 | 0.3 | 0.7 | 0 | 5 |  | 314 | 0.4 | 0.5 | 0 | 2 |
| Sons who have died | 13,612 | 0.2 | 0.6 | 0 | 6 |  | 314 | 0.1 | 0.3 | 0 | 1 |
| Daughters who have died | 13,612 | 0.2 | 0.5 | 0 | 6 |  | 314 | 0.3 | 0.4 | 0 | 1 |
| Births in last five years | 13,612 | 0.9 | 0.8 | 0 | 5 |  | 314 | 0.0 | 0.0 | 0 | 0 |
| Births in past year | 13,612 | 0.2 | 0.4 | 0 | 3 |  | 314 | 0.0 | 0.0 | 0 | 0 |
| Births in month of interview | 13,612 | 0.0 | 0.1 | 0 | 2 |  | 314 | 0.0 | 0.0 | 0 | 0 |
| Currently pregnant | 13,612 | 0.1 | 0.3 | 0 | 1 |  | 314 | 0.3 | 0.5 | 0 | 1 |
| Menstruated in last six weeks | 13,612 | 0.6 | 0.5 | 0 | 1 |  | 314 | 0.6 | 0.5 | 0 | 1 |
| Entries in birth history | 13,612 | 3.0 | 2.5 | 0 | 16 |  | 314 | 3.0 | 0.4 | 2 | 4 |
| Births in last three years | 13,612 | 0.5 | 0.6 | 0 | 4 |  | 314 | 0.0 | 0.0 | 0 | 0 |
| Ever used anything or tried to delay or avoid getting pregnant | 13,612 | 1.1 | 1.0 | 0 | 2 |  | 314 | 0.9 | 0.8 | 0 | 2 |
| Current contraceptive method | 13,612 | 0.1 | 0.2 | 0 | 1 |  | 314 | 0.1 | 0.2 | 0 | 1 |
| Current contraceptive by method type | 13,612 | 1.2 | 1.5 | 0 | 3 |  | 314 | 1.3 | 1.4 | 0 | 3 |
| Fecund (definition 3) | 13,612 | 0.6 | 0.5 | 0 | 1 |  | 314 | 0.6 | 0.5 | 0 | 1 |
| Household has: telephone (land-line) | 13,634 | 0.0 | 0.2 | 0 | 1 |  | 292 | 0.0 | 0.0 | 0 | 0 |
| Household has: electricity | 13,637 | 0.3 | 0.5 | 0 | 1 |  | 289 | 0.4 | 0.5 | 0 | 1 |
| Household has: television | 13,646 | 0.3 | 0.5 | 0 | 1 |  | 280 | 0.4 | 0.5 | 0 | 1 |
| Household has: refrigerator | 13,646 | 0.2 | 0.4 | 0 | 1 |  | 280 | 0.3 | 0.4 | 0 | 1 |
| Household has: motorcycle/scooter | 13,646 | 0.0 | 0.2 | 0 | 1 |  | 280 | 0.0 | 0.0 | 0 | 0 |
| Household has: car/truck | 13,646 | 0.1 | 0.3 | 0 | 1 |  | 280 | 0.0 | 0.1 | 0 | 1 |
| Household has: bicycle | 13,647 | 0.2 | 0.4 | 0 | 1 |  | 279 | 0.2 | 0.4 | 0 | 1 |
| Household has: radio | 13,648 | 0.5 | 0.5 | 0 | 1 |  | 278 | 0.6 | 0.5 | 0 | 1 |
| Currently breastfeeding | 13,756 | 0.3 | 0.5 | 0 | 1 |  | 170 | 0.0 | 0.1 | 0 | 1 |
| Total lifetime number of sex partners | 13,846 | 2.0 | 4.8 | 1 | 146 |  | 80 | 3.4 | 2.5 | 1 | 11 |
| Had genital sore/ulcer in last 12 months | 13,859 | 0.0 | 0.2 | 0 | 1 |  | 67 | 0.0 | 0.0 | 0 | 0 |
| Had any STI in last 12 months | 13,860 | 0.0 | 0.2 | 0 | 1 |  | 66 | 0.0 | 0.0 | 0 | 0 |
| Had genital discharge in last 12 months | 13,862 | 0.1 | 0.2 | 0 | 1 |  | 64 | 0.0 | 0.0 | 0 | 0 |
| Recent sexual activity | 13,907 | 0.6 | 0.5 | 0 | 1 |  | 19 | 0.3 | 0.5 | 0 | 1 |
| Currently working | 13,909 | 0.6 | 0.5 | 0 | 1 |  | 17 | 0.0 | 0.0 | 0 | 0 |
| Frequency of reading newspaper or magazine | 13,911 | 0.4 | 0.7 | 0 | 3 |  | 15 | 0.5 | 0.5 | 0 | 1 |
| Number of sex partners, including spouse, in last 12 months | 13,911 | 0.9 | 0.6 | 0 | 50 |  | 15 | 0.5 | 0.5 | 0 | 1 |
| Heard about other STIs | 13,911 | 0.7 | 0.4 | 0 | 1 |  | 15 | 1.0 | 0.0 | 1 | 1 |
| Cluster altitude in meters | 13,912 | 1,237.5 | 630.1 | 1 | 3,455 |  | 14 | 672.4 | 141.6 | 373 | 853 |
| Time since last sex (in days) | 13,912 | 274.4 | 946.0 | 0 | 32,850 |  | 14 | 441.9 | 409.5 | 0 | 1,149 |
| Ever been tested for HIV | 13,913 | 0.8 | 0.4 | 0 | 1 |  | 13 | 0.9 | 0.3 | 0 | 1 |
| Age of household head | 13,915 | 41.0 | 13.3 | 15 | 100 |  | 11 | 43.9 | 13.8 | 21 | 64 |
| Frequency of watching television | 13,919 | 0.7 | 1.1 | 0 | 3 |  | 7 | 0.0 | 0.0 | 0 | 0 |
| Religion_catholicism | 13,919 | 0.2 | 0.4 | 0 | 1 |  | 7 | 0.0 | 0.0 | 0 | 0 |
| Religion_islam | 13,919 | 0.1 | 0.3 | 0 | 1 |  | 7 | 0.0 | 0.0 | 0 | 0 |
| Religion_no religion | 13,919 | 0.0 | 0.1 | 0 | 1 |  | 7 | 0.0 | 0.0 | 0 | 0 |
| Religion_other | 13,919 | 0.1 | 0.3 | 0 | 1 |  | 7 | 0.1 | 0.4 | 0 | 1 |
| Religion_other christian | 13,919 | 0.1 | 0.3 | 0 | 1 |  | 7 | 0.3 | 0.5 | 0 | 1 |
| Religion_protestantism | 13,919 | 0.5 | 0.5 | 0 | 1 |  | 7 | 0.6 | 0.5 | 0 | 1 |
| Total number of years of education | 13,921 | 5.6 | 4.4 | 0 | 21 |  | 5 | 4.0 | 2.4 | 1 | 7 |
| Frequency of listening to radio | 13,922 | 1.0 | 1.0 | 0 | 3 |  | 4 | 1.8 | 0.5 | 1 | 2 |
| Number of injections in last 12 months | 13,922 | 1.3 | 5.3 | 0 | 146 |  | 4 | 2.3 | 2.6 | 0 | 6 |
| Presence of other people for 'Wife beating justified' questions | 13,922 | 0.1 | 0.3 | 0 | 1 |  | 4 | 0.0 | 0.0 | 0 | 0 |
| Highest educational level | 13,923 | 1.1 | 0.8 | 0 | 3 |  | 3 | 0.7 | 0.6 | 0 | 1 |
| Presence of other people during the sexual activity section of the interview | 13,923 | 0.0 | 0.2 | 0 | 1 |  | 3 | 0.0 | 0.0 | 0 | 0 |
| Usual resident or visitor | 13,924 | 1.0 | 0.1 | 0 | 1 |  | 2 | 1.0 | 0.0 | 1 | 1 |
| Wife justified asking husband to use condom if he has STI | 13,924 | 0.8 | 0.4 | 0 | 1 |  | 2 | 1.0 | 0.0 | 1 | 1 |
| Respondent worked in last 7 days | 13,925 | 1.7 | 1.4 | 0 | 3 |  | 1 | 0.0 |  | 0 | 0 |
| Beating justified | 13,925 | 0.4 | 0.5 | 0 | 1 |  | 1 | 1.0 |  | 1 | 1 |
