# Supplementary material for: Prediction of HIV status based on socio-behavioural characteristics in East and Southern Africa
Source: PLoS One. 2022 Mar 3;17(3):e0264429. doi: 10.1371/journal.pone.0264429 (PMC8893684; doi:10.1371/journal.pone.0264429)
Supplement: S5 Table — (DOCX) [file pone.0264429.s007.docx]

**Table S4: List of variables**

Variable names correspond to the name in the Demographic and Health Survey (DHS).

| **Common variables for males and females** | |
| --- | --- |
| Age at first sex (imputed) | Ideal number of either sex |
| Age of household head | Ideal number of girls |
| Age of most recent partner | Know a place to get HIV test |
| Beating justified | Knowledge of any contraceptive method |
| Cluster altitude in meters | Knowledge of ovulatory cycle |
| Cluster's latitude coordinate | Literacy |
| Cluster's longitude coordinate | Number of household members (total listed) |
| Cohabitation duration (grouped) | Number of injections in last 12 months |
| Condom used during last sex with most recent partner | Number of sex partners, including spouse, in last 12 months |
| Country | Occupation |
| Covered by health insurance | Owns a house alone or jointly |
| Current age | Owns land alone or jointly |
| Current contraceptive by method type | Recent sexual activity |
| Current contraceptive method | Reduce risk of getting HIV |
| Currently/formerly/never in union | Relationship to household head |
| Currently working | Relationship with most recent sex partner |
| Daughters at home | Religion |
| Daughters elsewhere | Respondent worked in last 7 days |
| Daughters who have died | Sex of household head |
| Drugs to avoid HIV transmission to baby during pregnancy | Sons at home |
| Ever been tested for HIV | Sons elsewhere |
| Ever heard of AIDS | Number of sons who have died |
| Ever heard of a Sexually Transmitted Infection (STI) | Time since last sex (in days) |
| Fertility preference | Time away from home in last 12 months |
| Frequency of listening to radio | Time in last 12 months had sex with most recent partner |
| Frequency of reading newspaper or magazine | Total lifetime number of sex partners |
| Frequency of watching television | Total number of years of education |
| Had any STI in last 12 months | Type of place of residence |
| Had genital discharge in last 12 months | Usual resident or visitor |
| Had genital sore/ulcer in last 12 months | Ways of transmission from mother to child |
| Heard about other STIs | Wealth index combined |
| Heard about family planning in newspaper/magazine during last few months | Wealth index factor score combined |
| Heard about family planning on radio during last few months | Wife justified asking husband to use condom if he has STI |
| Heard about family planning on TV during last few months | Wife justified refusing sex: husband has other women |
| Highest educational level | Would buy vegetables from vendor with HIV |
| Ideal number of boys | Years lived in place of residence |
| Ideal number of children |  |
| **Specific variables for females** | |
| Age at first cohabitation | Household has: electricity |
| Births in last five years | Household has: motorcycle/scooter |
| Births in last three years | Household has: radio |
| Births in month of interview | Household has: refrigerator |
| Births in past year | Household has: telephone (land-line) |
| Contraceptive use and intention | Household has: television |
| Currently abstaining | Index last child prior to maternity-health (calendar) |
| Currently amenorrhoeic | Menstruated in last six weeks |
| Currently breastfeeding | Number of children 5 and under in household (de jure) |
| Currently pregnant | Number of eligible women in household (de facto) |
| Does not use cigarettes and tobacco | Number of unions |
| Entries in birth history | Pattern of contraceptive use |
| Entries in immunization roster | Presence of other people during the sexual activity section of the interview |
| Entries in pregnancy and postnatal care roster | Presence of other people for 'Wife beating justified' questions |
| Ever had a terminated pregnancy | Record for Last Birth |
| Ever used anything or tried to delay or avoid getting pregnant | Respondent slept under mosquito bed net |
| Fecund (definition 3) | Rohrer's index |
| Getting medical help for self: distance to health facility | Time to get to water source |
| Getting medical help for self: getting money needed for treatment | Toilet facilities shared with other households |
| Getting medical help for self: getting permission to go | Type of mosquito bed net(s) slept under last night |
| Getting medical help for self: not wanting to go alone | Unmet need for contraception |
| Have mosquito bed net for sleeping | Visited by fieldworker in last 12 months |
| Heard of oral rehydration | Visited health facility last 12 months |
| Household has: bicycle | Years since first cohabitation |
| Household has: car/truck |  |
| **Specific variables for males** | |
| Contraception is woman's business, man should not worry | Number of women fathered children with |
| Discussed Family Planning with health worker in last few months | Paid for sex in last 12 months |
| Employment all year/seasonal | Respondent circumcised |
| Have ever paid anyone in exchange for sex | Type of earnings from respondent's work |
| Number of eligible men in household (de facto) | Women who use contraception become promiscuous |
| Number of wives/partners |  |
|  |  |
