# Supplementary material for: Prediction of HIV status based on socio-behavioural characteristics in East and Southern Africa
Source: PLoS One. 2022 Mar 3;17(3):e0264429. doi: 10.1371/journal.pone.0264429 (PMC8893684; doi:10.1371/journal.pone.0264429)
Supplement: S6 Table — (DOCX) [file pone.0264429.s008.docx]

**Table S5: Characteristics of Demographic and Health Survey (DHS) individuals**

|  | **Male** | | **Female** | |
| --- | --- | --- | --- | --- |
| **Total number of individuals** | 55,151 | | 69,626 | |
| **Current age, n (%)** |  | |  | |
| 15-24 | 14,472 (26.2%) | | 20,506 (29.5%) | |
| 25-34 | 17,584 (31.9%) | | 25,020 (35.9%) | |
| 35-44 | 13,074 (23.7%) | | 17,164 (24.7%) | |
| 45-54 | 7,853 (14.2%) | | 6,203 (8.9%) | |
| 55-64 | 2,168 (3.9%) | | 733 (1.1%) | |
| **Country of origin, n (%)** |  | |  | |
| Angola | 4,611 (8.4%) | | 6,020 (8.6%) | |
| Burundi | 5,180 (9.4%) | | 6,007 (8.6%) | |
| Ethiopia | 7,955 (14.4%) | | 11,162 (16.0%) | |
| Lesotho | 2,438 (4.4%) | | 2,917 (4.2%) | |
| Malawi | 5,657 (10.3%) | | 6,895 (9.9%) | |
| Mozambique | 4,053 (7.3%) | | 6,472 (9.3%) | |
| Namibia | 3,326 (6.0%) | | 4,431 (6.4%) | |
| Rwanda | 4,613 (8.4%) | | 4,948 (7.1%) | |
| Zambia | 11,617 (21.1%) | | 13,453 (19.3%) | |
| Zimbabwe | 5,701 (10.3%) | | 7,321 (10.5%) | |
| **HIV positive by country, n (% of individuals within country)** |  | |  | |
| Angola |  | 48 (1.0%) |  | 164 (2.7%) |
| Burundi |  | 49 (0.9%) |  | 92 (1.5%) |
| Ethiopia |  | 66 (0.8%) |  | 172 (1.5%) |
| Lesotho |  | 531 (21.8%) |  | 970 (33.3%) |
| Malawi |  | 445 (7.9%) |  | 837 (12.1%) |
| Mozambique |  | 434 (10.7%) |  | 1,002 (15.5%) |
| Namibia |  | 433 (13.0%) |  | 810 (18.3%) |
| Rwanda |  | 157 (3.4%) |  | 264 (5.3%) |
| Zambia |  | 1,494 (12.9%) |  | 2,235 (16.6%) |
| Zimbabwe |  | 760 (13.3%) |  | 1,465 (20.0%) |
| **Type of residence, n (%)** |  | |  | |
| Urban | 19,196 (34.8%) | | 23,501 (33.8%) | |
| Rural | 35,955 (65.2%) | | 46,125 (66.2%) | |
