# Supplementary material for: Prediction of HIV status based on socio-behavioural characteristics in East and Southern Africa
Source: PLoS One. 2022 Mar 3;17(3):e0264429. doi: 10.1371/journal.pone.0264429 (PMC8893684; doi:10.1371/journal.pone.0264429)
Supplement: S7 Table — (DOCX) [file pone.0264429.s009.docx]

**Table S6i: Results of the XGBoost algorithm per sex for the validation (80%; 5-fold cross-validation), test (20%) and, left-out (excluded country) samples**

|  |  | **Males** | | | | | **Females** | | | | |
| --- | --- | --- | --- | --- | --- | --- | --- | --- | --- | --- | --- |
| **Country** | **Metric** | f1 score | Sensitivity | PPV | Brier score | Prevalence | f1 score | Sensitivity | PPV | Brier score | Prevalence |
| Angola | Validation | 73·3% (± 2·3%) | 70·7% (± 3·7%) | 76·2% (± 2·4%) | 3·6% (± 0.4%) | 8·6% | 74·9% (± 0·8%) | 72·6% (± 1·5%) | 77·4% (± 0·4%) | 5·0% (± 0.2%) | 12·3% |
|  | Test | 75·9% | 72·2% | 80·1% | 3·1% | 8·6% | 78·9% | 76·1% | 81·9% | 4·0% | 12·3% |
|  | Left-out | 6·6% | 16·7% | 4·1% | 1.0% | 1.0% | 12·2% | 12·8% | 11·6% | 2.6% | 2·7% |
| Burundi | Validation | 73·8% (± 2·0%) | 70·7% (± 2·3%) | 77·3% (± 3·6%) | 3·6% (± 0.3%) | 8·7% | 74·5% (± 1·0%) | 74·5% (± 1·6%) | 74·6% (± 1·2%) | 5·0% (± 0.1%) | 12·4% |
|  | Test | 75·6% | 73·0% | 78·4% | 3·3% | 8·7% | 79·0% | 78·1% | 79·9% | 4·0% | 12·4% |
|  | Left-out | 17·1% | 14·3% | 21·2% | 0·9% | 0·9% | 17·6% | 22·8% | 14·3% | 1·5% | 1·5% |
| Ethiopia | Validation | 72·7% (± 2·4%) | 66·3% (± 4·0%) | 80·6% (± 1·3%) | 3·9% (± 0.2%) | 9·2% | 74·9% (± 0·6%) | 71·8% (± 1·0%) | 78·3% (± 1·1%) | 5·3% (± 0.4%) | 13·4% |
|  | Test | 78·9% | 74·8% | 83·4% | 3·1% | 9·2% | 80·0% | 78·1% | 81·9% | 4·3% | 13·4% |
|  | Left-out | 12·5% | 7·6% | 35·7% | 0·8% | 0·8% | 5·3% | 3·5% | 11·1% | 1·5% | 1·5% |
| Lesotho | Validation | 72·9% (± 1·7%) | 68·4% (± 3·6%) | 78·1% (± 2·3%) | 3·1% (± 0.2%) | 7·4% | 74·7% (± 1·1%) | 71·0% (± 1·7%) | 78·9% (± 1·5%) | 4·3% (± 0.3%) | 10·6% |
|  | Test | 76·4% | 71·9% | 81·4% | 2·6% | 7·4% | 78·0% | 74·8% | 81·6% | 3·7% | 10·6% |
|  | Left-out | 32·8% | 22·6% | 60·0% | 15·4% | 21·8% | 47·5% | 37·5% | 64·8% | 18·6% | 33·3% |
| Malawi | Validation | 73·4% (± 2·7%) | 69·5% (± 2·3%) | 77·7% (± 3·8%) | 3·3% (± 0.3%) | 8.0% | 75·9% (± 1·5%) | 73·2% (± 1·4%) | 78·9% (± 1·8%) | 4·5% (± 0.3%) | 11·4% |
|  | Test | 77·3% | 73·4% | 81·7% | 2.8% | 8.0% | 78·6% | 76·1% | 81·2% | 3.8% | 11·4% |
|  | Left-out | 24·9% | 18·9% | 36·7% | 6·7% | 7·9% | 32·8% | 30·1% | 36·1% | 9·8% | 12·1% |
| Mozambique | Validation | 74·1% (± 1·7%) | 68·3% (± 2·5%) | 81·0% (± 3·3%) | 3·2% (± 0.2%) | 7·8% | 76·6% (± 1·0%) | 75·4% (± 1·1%) | 78·0% (± 1·3%) | 4·2% (± 0.2%) | 11·1% |
|  | Test | 75·5% | 68·4% | 84·4% | 3·0% | 7·8% | 80·5% | 79·2% | 81·9% | 3·4% | 11·1% |
|  | Left-out | 14·4% | 9·9% | 26·4% | 9·4% | 10·7% | 31·2% | 25·8% | 39·2% | 12·1% | 15·5% |
| Namibia | Validation | 73·6% (± 1·7%) | 69·6% (± 1·1%) | 78·1% (± 3·3%) | 3·2% (± 0.2%) | 7·7% | 75·4% (± 1·2%) | 73·4% (± 1·0%) | 77·5% (± 1·4%) | 4·3% (± 0.2%) | 11.0% |
|  | Test | 77·4% | 73·8% | 81·3% | 2·6% | 7·7% | 78·2% | 77·8% | 78·6% | 3·6% | 11.0% |
|  | Left-out | 31·2% | 27·9% | 35·3% | 10.6% | 13.0% | 41·8% | 41·5% | 42·1% | 13.3% | 18·3% |
| Rwanda | Validation | 72·9% (± 2·5%) | 69·7% (± 2·7%) | 76·4% (± 2·6%) | 3·5% (± 0.3%) | 8·4% | 75·5% (± 1·1%) | 74·7% (± 1·7%) | 76·3% (± 1·1%) | 4·7% (± 0.2%) | 12.0% |
|  | Test | 78·0% | 75·6% | 80·6% | 2·8% | 8·4% | 79·3% | 79·7% | 78·9% | 3·8% | 12.0% |
|  | Left-out | 11·3% | 6·4% | 50·0% | 3·1% | 3·4% | 20·8% | 16·7% | 27·5% | 4·8% | 5·3% |
| Zambia | Validation | 72·8% (± 2·3%) | 69·4% (± 2·0%) | 76·6% (± 3·5%) | 2·8% (± 0.2%) | 6·7% | 74·2% (± 2·1%) | 73·7% (± 1·9%) | 74·8% (± 2·6%) | 4·2% (± 0.3%) | 10·3% |
|  | Test | 76·7% | 73·0% | 80·7% | 2·3% | 6·7% | 77·7% | 77·0% | 78·5% | 3·5% | 10·3% |
|  | Left-out | 22·3% | 14·1% | 53·0% | 10·4% | 12·9% | 41·5% | 37·1% | 47·0% | 12·1% | 16·6% |
| Zimbabwe | Validation | 73·2% (± 1·4%) | 66·9% (± 1·6%) | 80·7% (± 1·6%) | 3·1% (± 0.2%) | 7·4% | 75·0% (± 0·7%) | 73·1% (± 1·6%) | 77·1% (± 1·1%) | 4·1% (± 0.1%) | 10·5% |
|  | Test | 76·3% | 70·7% | 82·9% | 2·5% | 7·4% | 78·2% | 77·4% | 79·0% | 3·5% | 10·5% |
|  | Left-out | 31·3% | 27·2% | 36·9% | 10·8% | 13·3% | 47·0% | 41·3% | 54·5% | 13·3% | 20.0% |

Positive Predictive Value (PPV)

(± %): 95% Confidence Interval
