# Supplementary material for: Prediction of HIV status based on socio-behavioural characteristics in East and Southern Africa
Source: PLoS One. 2022 Mar 3;17(3):e0264429. doi: 10.1371/journal.pone.0264429 (PMC8893684; doi:10.1371/journal.pone.0264429)
Supplement: S8 Table — (DOCX) [file pone.0264429.s010.docx]

**Table S6ii: Results of the Support Vector Machine (SVM) algorithm per sex for the validation, test and, left-out samples**

|  |  | **Males** | | | | | **Females** | | | | |
| --- | --- | --- | --- | --- | --- | --- | --- | --- | --- | --- | --- |
| **Country** | **Metric** | f1 score | Sensitivity | PPV | Brier score | Prevalence | f1 score | Sensitivity | PPV | Brier score | Prevalence |
| Angola | Validation | 64·1% (± 2·2%) | 66·4% (± 2·0%) | 62·0% (± 2·9%) | 5·3% (± 0.2%) | 8·6% | 70·3% (± 1·2%) | 69·3% (± 1·1%) | 71·4% (± 1·8%) | 5·6% (± 0.1%) | 12·3% |
|  | Test | 70·1% | 71·6% | 68·7% | 4·6% | 8·6% | 74·3% | 73·3% | 75·3% | 4·9% | 12·3% |
|  | Left-out | 10·2% | 22·9% | 6·5% | 1.0% | 1.0% | 4·9% | 7·9% | 3·5% | 2.6% | 2·7% |
| Burundi | Validation | 64·9% (± 0·6%) | 66·4% (± 1·6%) | 63·6% (± 2·6%) | 4·9% (± 0.1%) | 8·7% | 71·0% (± 1·2%) | 68·5% (± 1·2%) | 73·7% (± 2·8%) | 5·3% (± 0.2%) | 12·4% |
|  | Test | 68·5% | 69·5% | 67·5% | 4·3% | 8·7% | 75·3% | 72·1% | 78·9% | 4·6% | 12·4% |
|  | Left-out | 11·2% | 22·4% | 7·4% | 0·9% | 0·9% | 8·0% | 12·0% | 6·0% | 1·5% | 1·5% |
| Ethiopia | Validation | 63·8% (± 2·7%) | 64·5% (± 3·9%) | 63·1% (± 2·4%) | 5·5% (± 0.2%) | 9·2% | 70·1% (± 1·2%) | 67·7% (± 1·1%) | 72·8% (± 1·8%) | 5·9% (± 0.2%) | 13·4% |
|  | Test | 70·4% | 73·2% | 67·8% | 4·6% | 9·2% | 75·1% | 74·4% | 75·8% | 5·0% | 13·4% |
|  | Left-out | 3·2% | 7·6% | 2·0% | 0·8% | 0·8% | 18·9% | 23·8% | 15·6% | 1·4% | 1·5% |
| Lesotho | Validation | 64·5% (± 0·9%) | 65·6% (± 2·2%) | 63·5% (± 2·0%) | 4·3% (± 0.1%) | 7·4% | 69·7% (± 1·3%) | 67·4% (± 1·6%) | 72·1% (± 1·9%) | 4·7% (± 0.1%) | 10·6% |
|  | Test | 69·4% | 70·5% | 68·3% | 3·7% | 7·4% | 73·3% | 71·2% | 75·4% | 4·1% | 10·6% |
|  | Left-out | 22·3% | 15·4% | 39·8% | 16·2% | 21·8% | 43·9% | 37·3% | 53·3% | 20·3% | 33·3% |
| Malawi | Validation | 65·2% (± 2·3%) | 65·5% (± 3·3%) | 65·0% (± 1·5%) | 4·5% (± 0.2%) | 8.0% | 70·8% (± 1·2%) | 68·8% (± 1·5%) | 72·9% (± 1·2%) | 4·9% (± 0.2%) | 11·4% |
|  | Test | 69·9% | 70·5% | 69·2% | 3.9% | 8.0% | 75·0% | 73·0% | 77·1% | 4.2% | 11·4% |
|  | Left-out | 21·7% | 17·8% | 27·8% | 7·0% | 7·9% | 25·9% | 21·7% | 32·0% | 10·0% | 12·1% |
| Mozambique | Validation | 66·6% (± 1·2%) | 67·7% (± 1·7%) | 65·6% (± 1·5%) | 4·4% (± 0.1%) | 7·8% | 72·4% (± 1·9%) | 70·2% (± 2·1%) | 74·7% (± 2·3%) | 4·5% (± 0.3%) | 11·1% |
|  | Test | 68·7% | 69·8% | 67·7% | 4·0% | 7·8% | 77·1% | 74·2% | 80·2% | 3·8% | 11·1% |
|  | Left-out | 11·4% | 8·5% | 17·4% | 9·4% | 10·7% | 24·9% | 21·6% | 29·5% | 12·5% | 15·5% |
| Namibia | Validation | 66·4% (± 1·5%) | 66·8% (± 3·1%) | 66·0% (± 0·6%) | 4·3% (± 0.1%) | 7·7% | 70·1% (± 1·9%) | 67·5% (± 2·1%) | 72·8% (± 2·1%) | 4·8% (± 0.2%) | 11.0% |
|  | Test | 69·8% | 70·1% | 69·5% | 3·9% | 7·7% | 74·1% | 71·7% | 76·7% | 4·1% | 11.0% |
|  | Left-out | 15·5% | 11·1% | 25·9% | 10.9% | 13.0% | 29·6% | 27·8% | 31·7% | 14.3% | 18·3% |
| Rwanda | Validation | 65·2% (± 2·4%) | 65·9% (± 3·0%) | 64·6% (± 2·7%) | 4·8% (± 0.1%) | 8·4% | 70·8% (± 1·2%) | 68·7% (± 1·4%) | 73·1% (± 2·0%) | 5·1% (± 0.2%) | 12.0% |
|  | Test | 71·1% | 73·6% | 68·8% | 4·0% | 8·4% | 75·0% | 73·7% | 76·4% | 4·5% | 12.0% |
|  | Left-out | 19·5% | 22·9% | 17·0% | 3·2% | 3·4% | 15·8% | 13·6% | 18·8% | 5·5% | 5·3% |
| Zambia | Validation | 65·0% (± 2·2%) | 64·8% (± 2·0%) | 65·2% (± 2·4%) | 3·7% (± 0.1%) | 6·7% | 70·2% (± 1·5%) | 67·1% (± 1·6%) | 73·7% (± 2·2%) | 4·5% (± 0.2%) | 10·3% |
|  | Test | 66·9% | 67·2% | 66·6% | 3·3% | 6·7% | 74·0% | 71·0% | 77·4% | 3·9% | 10·3% |
|  | Left-out | 21·1% | 15·5% | 33·2% | 10·7% | 12·9% | 31·3% | 23·5% | 47·0% | 12·5% | 16·6% |
| Zimbabwe | Validation | 63·7% (± 1·8%) | 64·5% (± 1·9%) | 62·8% (± 1·9%) | 4·3% (± 0.1%) | 7·4% | 70·4% (± 1·0%) | 67·8% (± 2·1%) | 73·2% (± 2·1%) | 4·6% (± 0.0%) | 10·5% |
|  | Test | 67·3% | 67·6% | 66·9% | 3·7% | 7·4% | 72·6% | 70·1% | 75·3% | 4·2% | 10·5% |
|  | Left-out | 18·3% | 14·6% | 24·4% | 11·4% | 13·3% | 19·7% | 12·5% | 46·8% | 15·2% | 20.0% |

Positive Predictive Value (PPV)

(± %): 95% Confidence Interval
