# Supplementary material for: Prediction of HIV status based on socio-behavioural characteristics in East and Southern Africa
Source: PLoS One. 2022 Mar 3;17(3):e0264429. doi: 10.1371/journal.pone.0264429 (PMC8893684; doi:10.1371/journal.pone.0264429)
Supplement: S9 Table — (DOCX) [file pone.0264429.s011.docx]

**Table S6iii: Results of the ElasticNet algorithm per sex for the validation, test and, left-out samples**

|  |  | **Males** | | | | | **Females** | | | | |
| --- | --- | --- | --- | --- | --- | --- | --- | --- | --- | --- | --- |
| **Country** | **Metric** | f1 score | Sensitivity | PPV | Brier score | Prevalence | f1 score | Sensitivity | PPV | Brier score | Prevalence |
| Angola | Validation | 33·3% (± 1·6%) | 76·8% (± 3·4%) | 21·2% (± 1·1%) | 17.2% (± 0.5%) | 8·6% | 42·2% (± 0·7%) | 77·0% (± 1·6%) | 29·0% (± 0·5%) | 16·9% (± 0.2%) | 12·3% |
|  | Test | 33·7% | 77·6% | 21·5% | 17·0% | 8·6% | 43·1% | 77·8% | 29·8% | 16·6% | 12·3% |
|  | Left-out | 3·5% | 56·2% | 1·8% | 25.4% | 1.0% | 10·1% | 65·2% | 5·5% | 20.4% | 2·7% |
| Burundi | Validation | 33·7% (± 1·0%) | 76·8% (± 3·3%) | 21·6% (± 0·6%) | 17.2% (± 0.2%) | 8·7% | 42·1% (± 1·8%) | 76·4% (± 2·7%) | 29·1% (± 1·4%) | 17·1% (± 0.6%) | 12·4% |
|  | Test | 33·1% | 77·1% | 21·1% | 17.4% | 8·7% | 41·8% | 76·2% | 28·8% | 17·2% | 12·4% |
|  | Left-out | 6·8% | 63·3% | 3·6% | 13·7% | 0·9% | 13·1% | 75·0% | 7·2% | 12·5% | 1·5% |
| Ethiopia | Validation | 34·4% (± 0·7%) | 75·3% (± 1·6%) | 22·3% (± 0·6%) | 17·5% (± 0.4%) | 9·2% | 43·2% (± 1·3%) | 75·0% (± 2·4%) | 30·3% (± 1·0%) | 17·5% (± 0.2%) | 13·4% |
|  | Test | 33·3% | 72·2% | 21·6% | 17·5% | 9·2% | 43·6% | 75·9% | 30·6% | 17·3% | 13·4% |
|  | Left-out | 4·7% | 47·0% | 2·5% | 12·9% | 0·8% | 19·2% | 57·6% | 11·5% | 6·8% | 1·5% |
| Lesotho | Validation | 30·3% (± 0·9%) | 78·1% (± 2·2%) | 18·8% (± 0·5%) | 17·0% (± 0.2%) | 7·4% | 38·8% (± 0·8%) | 77·7% (± 1·7%) | 25·9% (± 0·6%) | 16·9% (± 0.3%) | 10·6% |
|  | Test | 29·8% | 77·3% | 18·5% | 17·0% | 7·4% | 38·6% | 78·1% | 25·6% | 16.9% | 10·6% |
|  | Left-out | 37·0% | 30·7% | 46·7% | 15·3% | 21·8% | 60·7% | 85·7% | 46·9% | 23·8% | 33·3% |
| Malawi | Validation | 32·9% (± 0·6%) | 79·1% (± 1·1%) | 20·8% (± 0·5%) | 16·6% (± 0.4%) | 8.0% | 41·9% (± 1·6%) | 78·9% (± 2·6%) | 28·5% (± 1·2%) | 16·3% (± 0.2%) | 11·4% |
|  | Test | 33·2% | 80·5% | 20·9% | 16.7% | 8.0% | 41·9% | 79·0% | 28·5% | 16.3% | 11·4% |
|  | Left-out | 31·1% | 49·9% | 22·6% | 13·4% | 7·9% | 37·8% | 58·9% | 27·8% | 16·4% | 12·1% |
| Mozambique | Validation | 32·4% (± 0·4%) | 78·5% (± 1·3%) | 20·4% (± 0·2%) | 16.3% (± 0.2%) | 7·8% | 42·2% (± 0·6%) | 79·1% (± 1·6%) | 28·8% (± 0·5%) | 15·9% (± 0.2%) | 11·1% |
|  | Test | 33·9% | 78·7% | 21·6% | 15·8% | 7·8% | 42·2% | 79·7% | 28·7% | 16·0% | 11·1% |
|  | Left-out | 23·6% | 29·0% | 19·9% | 14·6% | 10·7% | 30·1% | 80·5% | 18·5% | 45·1% | 15·5% |
| Namibia | Validation | 32·2% (± 0·6%) | 78·4% (± 1·4%) | 20·3% (± 0·5%) | 16·4% (± 0.4%) | 7·7% | 41·6% (± 1·3%) | 79·0% (± 1·0%) | 28·2% (± 1·1%) | 16·2% (± 0.6%) | 11.0% |
|  | Test | 32·3% | 76·9% | 20·4% | 16·3% | 7·7% | 41·0% | 78·0% | 27·8% | 16·4% | 11.0% |
|  | Left-out | 32·2% | 59·8% | 22·0% | 21.2% | 13.0% | 37·2% | 83·1% | 24·0% | 30.6% | 18·3% |
| Rwanda | Validation | 32·9% (± 0·6%) | 78·3% (± 2·0%) | 20·8% (± 0·4%) | 17·1% (± 0.3%) | 8·4% | 41·9% (± 1·0%) | 78·1% (± 1·1%) | 28·6% (± 0·9%) | 16·8% (± 0.5%) | 12.0% |
|  | Test | 32·7% | 78·5% | 20·7% | 17·2% | 8·4% | 43·0% | 80·1% | 29·4% | 16·7% | 12.0% |
|  | Left-out | 15·3% | 84·7% | 8·4% | 15·6% | 3·4% | 26·2% | 54·9% | 17·2% | 12·8% | 5·3% |
| Zambia | Validation | 31·7% (± 1·5%) | 80·5% (± 1·3%) | 19·8% (± 1·1%) | 15.7% (± 0.8%) | 6·7% | 40·5% (± 0·9%) | 80·0% (± 1·1%) | 27·1% (± 0·7%) | 15·9% (± 0.3%) | 10·3% |
|  | Test | 31·7% | 79·7% | 19·8% | 15·5% | 6·7% | 40·3% | 79·1% | 27·0% | 15·9% | 10·3% |
|  | Left-out | 32·5% | 70·5% | 21·2% | 24·7% | 12·9% | 43·8% | 72·8% | 31·4% | 21·0% | 16·6% |
| Zimbabwe | Validation | 31·1% (± 0·6%) | 78·2% (± 1·5%) | 19·4% (± 0·4%) | 16·7% (± 0.3%) | 7·4% | 39·3% (± 1·1%) | 78·1% (± 1·7%) | 26·3% (± 1·0%) | 16·4% (± 0.7%) | 10·5% |
|  | Test | 32·2% | 80·3% | 20·2% | 16·5% | 7·4% | 39·4% | 77·8% | 26·4% | 16·4% | 10·5% |
|  | Left-out | 27·1% | 63·4% | 17·2% | 32·1% | 13·3% | 47·8% | 68·2% | 36·8% | 19·7% | 20.0% |

Positive Predictive Value (PPV)

(± %): 95% Confidence Interval
