# Supplementary material for: Prediction of HIV status based on socio-behavioural characteristics in East and Southern Africa
Source: PLoS One. 2022 Mar 3;17(3):e0264429. doi: 10.1371/journal.pone.0264429 (PMC8893684; doi:10.1371/journal.pone.0264429)
Supplement: S10 Table — (DOCX) [file pone.0264429.s012.docx]

**Table S6iv: Results of the Generalized Additive Model (GAM) algorithm per sex for the validation, test and, left-out samples**

|  |  | **Males** | | | | | **Females** | | | | |
| --- | --- | --- | --- | --- | --- | --- | --- | --- | --- | --- | --- |
| **Country** | **Metric** | f1 score | Sensitivity | PPV | Brier score | Prevalence | f1 score | Sensitivity | PPV | Brier score | Prevalence |
| Angola | Validation | 25·5% (± 2·6%) | 16·4% (± 2·1%) | 57·6% (± 6·0%) | 6·4% (± 0.2%) | 8·6% | 39·8% (± 1·1%) | 29·0% (± 0·8%) | 63·5% (± 2·6%) | 8·1% (± 0.1%) | 12·3% |
|  | Test | 26·4% | 16·9% | 59·7% | 6·3% | 8·6% | 38·7% | 27·9% | 63·6% | 8·1% | 12·3% |
|  | Left-out | 0·0% | 0·0% | 0·0% | 1.1% | 1.0% | 4·4% | 2·4% | 21·1% | 2.9% | 2·7% |
| Burundi | Validation | 26·5% (± 2·8%) | 17·0% (± 2·2%) | 60·0% (± 4·6%) | 6·4% (± 0.1%) | 8·7% | 39·2% (± 2·6%) | 28·6% (± 2·6%) | 62·7% (± 2·6%) | 8·2% (± 0.1%) | 12·4% |
|  | Test | 24·1% | 15·0% | 61·2% | 6·5% | 8·7% | 37·9% | 27·3% | 62·2% | 8·3% | 12·4% |
|  | Left-out | 2·4% | 2·0% | 3·0% | 2·3% | 0·9% | 6·2% | 3·3% | 60·0% | 1·5% | 1·5% |
| Ethiopia | Validation | 26·1% (± 1·7%) | 16·9% (± 1·3%) | 58·0% (± 2·5%) | 6·8% (± 0.2%) | 9·2% | 39·4% (± 1·4%) | 28·7% (± 1·1%) | 62·8% (± 2·5%) | 8·9% (± 0.2%) | 13·4% |
|  | Test | 26·7% | 17·1% | 60·1% | 6·8% | 9·2% | 41·1% | 29·9% | 65·5% | 8·6% | 13·4% |
|  | Left-out | 0·0% | 0·0% | 0·0% | 3·8% | 0·8% | 0·0% | 0·0% | 0·0% | 1·6% | 1·5% |
| Lesotho | Validation | 22·8% (± 3·8%) | 14·2% (± 2·6%) | 57·7% (± 5·6%) | 5·6% (± 0.2%) | 7·4% | 34·5% (± 1·3%) | 24·1% (± 1·6%) | 61·4% (± 4·6%) | 7·3% (± 0.2%) | 10·6% |
|  | Test | 23·1% | 14·4% | 58·3% | 5·5% | 7·4% | 36·1% | 25·8% | 60·3% | 7·2% | 10·6% |
|  | Left-out | 12·6% | 13·9% | 11·5% | 39·1% | 21·8% | 47·5% | 37·5% | 64·5% | 18·1% | 33·3% |
| Malawi | Validation | 26·6% (± 4·3%) | 17·2% (± 3·2%) | 58·8% (± 5·6%) | 5·9% (± 0.2%) | 8.0% | 41·0% (± 1·4%) | 30·3% (± 1·4%) | 63·5% (± 1·9%) | 7·5% (± 0.2%) | 11·4% |
|  | Test | 28·5% | 18·3% | 64·4% | 5.8% | 8.0% | 41·9% | 30·5% | 67·2% | 7.4% | 11·4% |
|  | Left-out | 0·0% | 0·0% | 0·0% | 6·7% | 7·9% | 14·6% | 8·2% | 65·7% | 9·5% | 12·1% |
| Mozambique | Validation | 27·6% (± 3·3%) | 18·0% (± 2·4%) | 59·4% (± 4·8%) | 5·7% (± 0.1%) | 7·8% | 41·7% (± 1·7%) | 31·0% (± 1·5%) | 63·8% (± 1·8%) | 7·2% (± 0.1%) | 11·1% |
|  | Test | 25·5% | 16·1% | 62·1% | 5·7% | 7·8% | 43·3% | 32·1% | 66·6% | 7·1% | 11·1% |
|  | Left-out | 4·3% | 2·3% | 29·4% | 10·6% | 10·7% | 16·6% | 13·7% | 21·0% | 16·4% | 15·5% |
| Namibia | Validation | 26·4% (± 0·8%) | 17·0% (± 0·9%) | 59·8% (± 4·2%) | 5·7% (± 0.1%) | 7·7% | 39·0% (± 1·2%) | 28·3% (± 1·1%) | 62·9% (± 3·1%) | 7·4% (± 0.1%) | 11.0% |
|  | Test | 27·8% | 17·7% | 64·4% | 5·6% | 7·7% | 41·3% | 30·3% | 65·1% | 7·3% | 11.0% |
|  | Left-out | 8·9% | 5·1% | 34·9% | 11.0% | 13.0% | 17·0% | 9·9% | 61·1% | 14.1% | 18·3% |
| Rwanda | Validation | 25·2% (± 1·5%) | 16·1% (± 1·2%) | 58·8% (± 5·0%) | 6·3% (± 0.1%) | 8·4% | 40·1% (± 0·8%) | 29·3% (± 0·7%) | 63·3% (± 3·2%) | 7·9% (± 0.1%) | 12.0% |
|  | Test | 26·5% | 16·8% | 63·0% | 6·2% | 8·4% | 41·1% | 30·4% | 63·4% | 7·7% | 12.0% |
|  | Left-out | 0·0% | 0·0% | 0·0% | 3·1% | 3·4% | 5·9% | 3·4% | 21·4% | 4·9% | 5·3% |
| Zambia | Validation | 24·9% (± 2·3%) | 16·0% (± 2·0%) | 57·0% (± 5·8%) | 5·0% (± 0.1%) | 6·7% | 40·3% (± 2·8%) | 29·6% (± 2·6%) | 63·1% (± 2·0%) | 6·8% (± 0.1%) | 10·3% |
|  | Test | 27·9% | 17·9% | 62·9% | 4·9% | 6·7% | 39·0% | 28·3% | 62·5% | 6·9% | 10·3% |
|  | Left-out | 15·0% | 8·8% | 50·2% | 10·5% | 12·9% | 46·7% | 44·8% | 48·7% | 12·5% | 16·6% |
| Zimbabwe | Validation | 23·4% (± 1·5%) | 14·8% (± 1·2%) | 55·5% (± 2·8%) | 5·6% (± 0.1%) | 7·4% | 36·7% (± 1·7%) | 26·4% (± 1·2%) | 60·3% (± 3·3%) | 7·1% (± 0.1%) | 10·5% |
|  | Test | 26·0% | 16·4% | 62·5% | 5·4% | 7·4% | 37·0% | 26·3% | 62·7% | 7·1% | 10·5% |
|  | Left-out | 22·9% | 56·1% | 14·4% | 48·9% | 13·3% | 27·5% | 38·6% | 21·4% | 32·9% | 20.0% |

Positive Predictive Value (PPV)

(± %): 95% Confidence Interval
