# Supplementary material for: Prediction of HIV status based on socio-behavioural characteristics in East and Southern Africa
Source: PLoS One. 2022 Mar 3;17(3):e0264429. doi: 10.1371/journal.pone.0264429 (PMC8893684; doi:10.1371/journal.pone.0264429)
Supplement: S11 Table — (DOCX) [file pone.0264429.s013.docx]

**Table S7: F1, sensitivity, PPV and Brier score per country for models M4 and F4**

| **Test dataset** | **Male** | | | | **Female** | | | |
| --- | --- | --- | --- | --- | --- | --- | --- | --- |
|  |  |  |  |  |  |  |  |  |
|  | **F1** | **Sensitivity** | **PPV** | **Brier** | **F1** | **Sensitivity** | **PPV** | **Brier** |
|  |  |  |  |  |  |  |  |  |
| Angola | 81.8% | 69.2% | 100.0% | 0.5% | 61.8% | 51.5% | 77.3% | 1.6% |
|  |  |  |  |  |  |  |  |  |
| Burundi | 66.7% | 60.0% | 75.0% | 0.3% | 80.0% | 70.6% | 92.3% | 0.5% |
|  |  |  |  |  |  |  |  |  |
| Ethiopia | 81.5% | 68.8% | 100.0% | 0.4% | 71.9% | 62.2% | 85.2% | 0.7% |
|  |  |  |  |  |  |  |  |  |
| Lesotho | 81.6% | 76.6% | 87.2% | 6.3% | 79.9% | 79.3% | 80.5% | 10.2% |
|  |  |  |  |  |  |  |  |  |
| Malawi | 61.4% | 51.3% | 76.5% | 3.2% | 78.5% | 71.5% | 87.1% | 4.2% |
|  |  |  |  |  |  |  |  |  |
| Mozambique | 78.1% | 71.7% | 85.7% | 3.8% | 75.1% | 69.4% | 81.7% | 5.8% |
|  |  |  |  |  |  |  |  |  |
| Namibia | 73.9% | 68.0% | 81.0% | 4.8% | 79.5% | 75.7% | 83.8% | 6.4% |
|  |  |  |  |  |  |  |  |  |
| Rwanda | 63.8% | 53.6% | 78.9% | 1.7% | 73.6% | 69.6% | 78.0% | 1.8% |
|  |  |  |  |  |  |  |  |  |
| Zambia | 72.2% | 63.8% | 83.3% | 5.4% | 79.2% | 74.3% | 84.8% | 5.0% |
|  |  |  |  |  |  |  |  |  |
| Zimbabwe | 77.2% | 75.5% | 78.9% | 4.9% | 78.6% | 78.5% | 78.8% | 6.0% |
|  |  |  |  |  |  |  |  |  |
