# Supplementary material for: Prediction of HIV status based on socio-behavioural characteristics in East and Southern Africa
Source: PLoS One. 2022 Mar 3;17(3):e0264429. doi: 10.1371/journal.pone.0264429 (PMC8893684; doi:10.1371/journal.pone.0264429)
Supplement: S12 Table — (DOCX) [file pone.0264429.s014.docx]

**Table S8: Predicted Prevalence per Country**

| **Test dataset** | **Male** | | | | **Female** | | | |
| --- | --- | --- | --- | --- | --- | --- | --- | --- |
|  |  |  |  |  |  |  |  |  |
|  | **Prevalence** | **Predicted** | **Absolute** | **Relative** | **Prevalence** | **Predicted** | **Absolute** | **Relative** |
|  |  |  | **Difference** | **Difference** |  |  | **Difference** | **Difference** |
| Angola | 1.4% | 1.0% | -0.4% | -28.6% | 2.8% | 1.9% | -0.9% | -32.1% |
|  |  |  |  |  |  |  |  |  |
| Burundi | 0.5% | 0.4% | -0.1% | -20.0% | 1.4% | 1.1% | -0.3% | -21.4% |
|  |  |  |  |  |  |  |  |  |
| Ethiopia | 1.0% | 0.7% | -0.3% | -30.0% | 1.7% | 1.2% | -0.5% | -29.4% |
|  |  |  |  |  |  |  |  |  |
| Lesotho | 20.7% | 18.2% | -2.5% | -12.1% | 33.1% | 32.6% | -0.5% | -1.5% |
|  |  |  |  |  |  |  |  |  |
| Malawi | 6.6% | 4.4% | -2.2% | -33.3% | 13.1% | 10.8% | -2.3% | -17.6% |
|  |  |  |  |  |  |  |  |  |
| Mozambique | 11.2% | 9.4% | -1.8% | -16.1% | 16.1% | 13.7% | -2.4% | -14.9% |
|  |  |  |  |  |  |  |  |  |
| Namibia | 12.0% | 10.0% | -2.0% | -16.7% | 21.3% | 19.2% | -2.1% | -9.9% |
|  |  |  |  |  |  |  |  |  |
| Rwanda | 3.1% | 2.1% | -1.0% | -32.3% | 4.5% | 4.0% | -0.5% | -11.1% |
|  |  |  |  |  |  |  |  |  |
| Zambia | 13.2% | 10.1% | -3.1% | -23.5% | 15.9% | 13.9% | -2.0% | -12.6% |
|  |  |  |  |  |  |  |  |  |
| Zimbabwe | 13.9% | 13.3% | -0.6% | -4.3% | 18.1% | 18.0% | -0.1% | -0.6% |
|  |  |  |  |  |  |  |  |  |
