# Supplementary material for: Prediction of HIV status based on socio-behavioural characteristics in East and Southern Africa
Source: PLoS One. 2022 Mar 3;17(3):e0264429. doi: 10.1371/journal.pone.0264429 (PMC8893684; doi:10.1371/journal.pone.0264429)
Supplement: Attachment — Submitted filename: review_report.pdf [file pone.0264429.s015.pdf]

**Review report on PLoS ONE manuscript PONE-D-21-10249: E. Orel et al. "Prediction of HIV status based on socio-behavioural characteristics in East and Southern Africa"**

In this manuscript, the authors use publicly available socio-behavioural data from the Demographic and Health Surveys program for 10 countries in Eastern and Southern Africa to predict HIV status for individuals using machine learning and statistical learning models. The authors first compared four different types of model types (penalized logistic regression, generalized additive model, support vector machines  and gradient-boosted trees) to identify the best-performing type (gradient-boosted trees) according to the so-called F1 metric, which is a well-known but less transparent metric. Subsequently the authors then identified the subset of most contributing features and build new models of the best type around these. Based on these models, the authors then investigated two scenarios for targeting preventive action: a) the number of persons to be tested to reach a sensitivity of 95 % (true positive rate), and b) identified a population for which the probability of being HIV positive was higher than 95 %.

This is an area of significant importance, and indeed an area in which modern machine learning methods can be brought to bear for great societal impact. The paper is furthermore well-written and mostly easy to read, and the figures and tables are generally well-prepared (more on this below). It therefore saddens me to report, that I cannot support publication at this stage due to serious shortcomings of the methods description, and potentially also inconsistencies in the results which undermine the claims of the authors (this latter I cannot judge in full due to shortcomings of the methods description).

Major points

1) The methods section is missing a number of important aspects in order for me to assess in detail the steps the authors have taken.
   - How were the models fitted? What were the target variable, and what was the outcome from each of the trainings? As far as I can tell from the files shared in the author's model and code repository the model selection analysis is not included (and the readme in the repository is not providing much help). As will be apparent further below, details of model training will be important to assess the validity of the results the authors claim.
   - Description of the details of the imputation methods used are missing. Is the variance conserved for the MICE algorithm that the authors use? And what is the implications of using the imputation of the XGBoost algorithm? This needs to be clarified to know whether this step is artificially limiting or enhancing the presented results.
   - I cannot follow the details of the three-step training process (Fig. 1). Is the whole thing a nested cross-validation where the outer loop (Step 1) is across countries (switching "hold-out country") and the inner step (Steps 2 and 3) is the 5-fold cross-validation used during model training? I sense the approach is fine but cannot follow all steps to make sure.
   - Figures and tables describing data preparation and model training are largely missing captions. This makes it harder to follow the steps; in cases like Table A2 I simply cannot infer how to read the table. What does each row represent? What happens between each row?

2) Two of the four model types, specifically support vector machines and gradient-boosted trees, actually do not model the probability that an individual has HIV unless specific steps are taken (e.g. use Platt scaling). It does not seem to be the case that such steps have been taken but cannot know for sure due to insufficient methods details provided. It is highly problematic that gradient-boosted trees (the type which is selected by the authors) does not return probabilities

since the authors use it to identify the subpopulation which has more than 95 % probability of having HIV (scenario 2); put differently, the results from scenario 2 cannot be trusted since gradient-boosted trees are used. This concern seems to invalidate these results.

For context: These models assign individuals to classes based on classification rules (the sign of the solution to the convex quadratic optimization problem for support vector machines, the particular splitting rules used in the gradient-boosted trees (usually cross-entropy) [1]) instead of modeling directly the probability that an individual has HIV. These are examples of improper scoring rules which are well-known to cause incorrect probabilities. An observed probability can be inferred afterwards from these models, but that will depend on the particular elements of the dataset and as such will change with the addition of a single new datapoint, and is in general not a good estimate of the actual probability.

3) The results in Fig. 2 illustrates substantially lower F1 scores on the left-out samples for support vector machines and gradient-boosted trees (XGBoost) which needs to be investigated. This illustrates that the performance found in training is not at all retrieved when the models are applied to new data, i.e. it undermines the trust which can be put in the abilities of these models to offer usable predictions. This puts all presented results at risk of being incorrect! While the authors do observe this, no explanation is offered nor is further investigation conducted. As a minimum the authors would need to explain why this should not be a point of concern for trusting the results.

   It is unclear to me whether this is a result of overfitting, is a consequence of using the F1 metric (itself an improper scoring rule) to evaluate the models (that metric itself being a non-linear function of the class assignments of the models), or something else entirely.

4) It appears the authors are using the F1 score to pick the best model (in Algorithms, as part of Methods section). As already mentioned, this is an improper scoring rule (which does not indicate which model best predicts the probability that an individual has HIV) and selecting models based on this metric could lead to models with good F1 scores which are a bad representation of whether or not an individual has HIV! The authors will need to clarify that they are not selecting models based on an improper scoring rule.

   More details on considerations for the important topic of proper scoring rules for classification can be found in [2] and [3].

Minor points

1. The authors use random selection of model hyper-parameters, which is a well-established approach. It would be good with some graphical illustration of the improvement from hyper-parameter optimization as an insight into whether model type or hyperparameter tuning is more important for this case.
2. The authors use Shapley values to assess the impact of each covariate on the outcome, which is also an established approach. However, readers without deep statistical training could be led to believe that the type of impact suggested by Shapley values could be used for shaping intervention strategies. Unfortunately this would not be correct because Shapley values do not describe the causal impact of each covariate, only the additional change in overall outcome by adding this covariate. I would suggest to add a comment along these lines to mitigate any unintended conclusions.

[1] Hastie et al., *Elements of Statistical Learning*, 2nd Edition, Springer (2009)
[2] Chap 10 in Harrell, Jr., *Regression Modeling Strategies*, Springer (2015)
[3] Cross Validated discussion providing good introduction
https://stats.stackexchange.com/questions/312780/why-is-accuracy-not-the-best-measure-for-assessing-classification-models
