# Supplementary material for: Prediction of HIV status based on socio-behavioural characteristics in East and Southern Africa
Source: PLoS One. 2022 Mar 3;17(3):e0264429. doi: 10.1371/journal.pone.0264429 (PMC8893684; doi:10.1371/journal.pone.0264429)
Supplement: Attachment — Submitted filename: review.docx [file pone.0264429.s017.docx]

**Second review of “Prediction of HIV status based on socio-behavioral characteristics in East and Southern Africa” by E. Orel et al.**

I am happy to see that the reviewers have addressed most of my comments. In their reply, the provide satisfactory replies and they have made good changes to the manuscript, supplement and code repository which makes it easier to follow. I am also happy to see the new Fig 4 which is a great addition.

Unfortunately, there are still two outstanding points which I think should be further elaborated.

1) The authors have added description of the MICE imputation as I requested, but it is unfortunately not clear from this description if steps have been taken to ensure that the imputation does not affect the variance of the imputed variables. If no steps are taken, imputation can influence the distribution of the imputed variable (often reducing the variance, sometimes also affecting the mean), which will likely impact the results (e.g. influence the ability to provide accurate predictions). Judging from the first 10-15 rows in Table S3i it seems that the imputed variables have lower variance. The authors should clarify which steps have been taken to ensure that variance and mean have not been affected by the imputations.

2) The authors take several steps during their model identification and training procedure. This methodological rigor is a strong point of the paper. Unfortunately it can still be hard to follow all the steps the authors take, so I think it could improve the paper even more if the methods section started with an overview of the different steps. Furthermore, upon identifying the best model, the authors seem to follow a different process when fitting the selected model in order to draw the conclusions they present (using data from all 10 countries, fitting using a regular five-fold cross validation). I cannot seem to find a description of whether any special considerations have been made to the test sets for this model (which is used to generate the results in the paper) – are they randomly selected? Stratified? It would be good if the authors could comment on this as well.

I remain of the opinion that this is an interesting paper presenting a useful application of data driven methods for societal health. Conditional on the authors providing answers to the two points above I would be happy to recommend publication of this paper in PLoS ONE.
