# Supplementary material for: Prediction of HIV status based on socio-behavioural characteristics in East and Southern Africa
Source: PLoS One. 2022 Mar 3;17(3):e0264429. doi: 10.1371/journal.pone.0264429 (PMC8893684; doi:10.1371/journal.pone.0264429)
Supplement: Attachment — Submitted filename: Response to Reviewers.docx [file pone.0264429.s018.docx]

PONE-D-21-10249R1

Prediction of HIV status based on socio-behavioural characteristics in East and Southern Africa

PLOS ONE

Dear editor,

Many thanks for the helpful second reviews which allowed us to further improve the manuscript. Please find below our answers to the referee comments.

Yours sincerely,

Erol Orel, Aziza Merzouki and Olivia Keiser on behalf of all co-authors.

**Comments**

**Editorial Comments to Authors**

Please review your reference list to ensure that it is complete and correct. If you have cited papers that have been retracted, please include the rationale for doing so in the manuscript text, or remove these references and replace them with relevant current references. Any changes to the reference list should be mentioned in the rebuttal letter that accompanies your revised manuscript. If you need to cite a retracted article, indicate the article’s retracted status in the References list and also include a citation and full reference for the retraction notice.

The reference list has been reviewed and the list is complete and correct. No cited papers have been retracted. Two additional papers have been added to the reference list:

- 21 Jakobsen, J.C., Gluud, C., Wetterslev, J. et al. When and how should multiple imputation be used for handling missing data in randomised clinical trials – a practical guide with flowcharts. BMC Med Res Methodol 17, 162 (2017). <https://doi.org/10.1186/s12874-017-0442-1>
- 37 Nguyen, C.D., Carlin, J.B. & Lee, K.J. Model checking in multiple imputation: an overview and case study. Emerg Themes Epidemiol 14, 8 (2017). <https://doi.org/10.1186/s12982-017-0062-6>

While revising your submission, please upload your figure files to the Preflight Analysis and Conversion Engine (PACE) digital diagnostic tool, https://pacev2.apexcovantage.com/. PACE helps ensure that figures meet PLOS requirements. To use PACE, you must first register as a user. Registration is free. Then, login and navigate to the UPLOAD tab, where you will find detailed instructions on how to use the tool.

All the figures have been uploaded to the PACE digital diagnostic tool and have been modified accordingly to meet PLOS requirements.

**Reviewer 1**

I am happy to see that the reviewers have addressed most of my comments. In their reply, they provide satisfactory replies and they have made good changes to the manuscript, supplement and code repository which makes it easier to follow. I am also happy to see the new Fig 4 which is a great addition.

Unfortunately, there are still two outstanding points which I think should be further elaborated.

1) The authors have added description of the MICE imputation as I requested, but it is unfortunately not clear from this description if steps have been taken to ensure that the imputation does not affect the variance of the imputed variables. If no steps are taken, imputation can influence the distribution of the imputed variable (often reducing the variance, sometimes also affecting the mean), which will likely impact the results (e.g. influence the ability to provide accurate predictions). Judging from the first 10-15 rows in Table S3i it seems that the imputed variables have lower variance. The authors should clarify which steps have been taken to ensure that variance and mean have not been affected by the imputations.

Many thanks, we are happy that the important work done to address the comments and revise the paper is acknowledged and valued. Regarding MICE imputation, imputing missing data has always been a challenge, and as stated in the reviewer’s comment, results of any statistical analysis can be only as good as the quality of the data (garbage in - garbage out) and this is particularly true in presence of missing data. In our specific case, we have assumed that the missing values were Missing At Random (MAR) and used the MICE imputation method (similar to the flowchart below from Jakobsen, J.C., Gluud, C., Wetterslev, J. et al. When and how should multiple imputation be used for handling missing data in randomised clinical trials – a practical guide with flowcharts. BMC Med Res Methodol 17, 162 (2017). <https://doi.org/10.1186/s12874-017-0442-1>). We now explicitly mention this assumption in the revised manuscript (line 129) and cite the additional reference paper from Jakobsen et al.

In other words, we assumed that there might be a relationship between the propensity of missing values and the observed data. MAR data is more common than Missing Completely At Random (MCAR) in all disciplines. Where in the case of MCAR, missing and observed observations are generated from the same distribution, in the case of MAR, the missing and observed observations are no longer coming from the same distribution. Nguyen et al. [Nguyen, C.D., Carlin, J.B. & Lee, K.J. Model checking in multiple imputation: an overview and case study. Emerg Themes Epidemiol 14, 8 (2017). <https://doi.org/10.1186/s12982-017-0062-6>] suggests that these discrepancies between observed and imputed data are not necessarily problematic, since under MAR we may expect such differences to arise.

In order to check the obtained models in MICE and to ensure the validity of our findings, we have taken a multifaceted approach:

- We assessed the plausibility of the imputed data using subject matter knowledge, harmonizing imputed data to the observed data types (e.g. dichotomic) and to the domain of definition or scale (e.g. strictly positive or maximum value). For a complete overview, the python script can be found in the project repository at “scripts/data_processing_engineering/imputation.py”. This reference to the code was added to the Supplementary Material in the MICE imputation section.
- We compared the imputed data with the observed data to identify major problems with the imputation model. As a rule of thumb, Stuart et al. [Stuart EA, Azur M, Frangakis C, Leaf P. Multiple Imputation with large data sets: a case study of the children’s mental health initiative. Am J Epidemiol. 2009;169(9):1133–9.] proposed comparing the means and variances of observed and imputed values and suggested flagging variables if the ratio of variances of the observed and imputed values was less than 0.5 or greater than 2, or if the absolute difference in means was greater than two standard deviations.
- We compared the scores and results obtained with the MICE imputation method to the ones obtained using the XGBoost built-in method and found very similar values. This is presented in main manuscript line 241-248.
- We used MICE imputation in the first part of our analysis, when we compared the predictive performance of the different algorithms. The performance of all algorithms was compared over the same potentially imperfect imputed datasets. We did not use the MICE imputed datasets in the second part of our analysis, i.e. for variables selection and HIV status prediction (used XGBoost built-in method).

Finally, we have added as a limitation, the following sentence line 376-378 of the “Discussion” section: “Additionally, missing values were to be found in the data and implied making assumptions about their randomness and using imputation methods that are necessarily imperfect by nature.”

2) The authors take several steps during their model identification and training procedure. This methodological rigor is a strong point of the paper. Unfortunately, it can still be hard to follow all the steps the authors take, so I think it could improve the paper even more if the methods section started with an overview of the different steps.

For better clarity, we have rewritten the different steps for model identification and training procedure

in the “Methods” section by adding a specific sub-section named “Training, validation and test procedure steps” starting line 23.

Furthermore, upon identifying the best model, the authors seem to follow a different process when fitting the selected model in order to draw the conclusions they present (using data from all 10 countries, fitting using a regular five-fold cross validation). I cannot seem to find a description of whether any special considerations have been made to the test sets for this model (which is used to generate the results in the paper) – are they randomly selected? Stratified? It would be good if the authors could comment on this as well.

Thank you for pointing this out. We used the exact same training, validation and testing strategy than in the first part of our analysis except that no country was left out. We have made it more explicit in the “Methods” section starting line 178-181 (beginning of the sub-section “Variables selection and HIV status prediction”):

“For variables selection and HIV status prediction, we used the exact same training, validation and testing strategy than in the first part of our analysis except that no country was left out. We split each unique dataset par sex into a stratified 80% training and validation set and a 20% test set. The best algorithm was trained and validated using a random grid-search over 250 sets of hyperparameters and a stratified 5-fold cross-validation”.
